# Virologic effects of broadly neutralizing antibodies VRC01LS and VRC07-523LS on chronic HIV-1 infection

**DOI:** 10.1172/jci.insight.181496

**Published:** 2025-02-24

**Authors:** Myra Happe, Rebecca M. Lynch, Carl J. Fichtenbaum, Sonya L. Heath, Susan L. Koletar, Raphael J. Landovitz, Rachel M. Presti, Jorge L. Santana-Bagur, Randall L. Tressler, LaSonji A. Holman, Laura Novik, Jhoanna C. Roa, Ro Shauna Rothwell, Larisa Strom, Jing Wang, Zonghui Hu, Michelle Conan-Cibotti, Anjali M. Bhatnagar, Bridget Dwyer, Sung Hee Ko, Frida Belinky, Aryan M. Namboodiri, Janardan P. Pandey, Robin Carroll, Manjula Basappa, Leonid Serebryannyy, Sandeep R. Narpala, Bob C. Lin, Adrian B. McDermott, Eli A. Boritz, Edmund V. Capparelli, Emily E. Coates, Richard A. Koup, Julie E. Ledgerwood, John R. Mascola, Grace L. Chen, Pablo Tebas, the VRC 607/A5378 Study Team

**Affiliations:** 1 Vaccine Research Center, National Institute of Allergy and Infectious Diseases, NIH, Bethesda, Maryland, USA.; 2 Department of Microbiology, Immunology, and Tropical Medicine, The George Washington University, Washington, DC, USA.; 3 Division of Infectious Diseases, University of Cincinnati, Ohio, USA.; 4 Division of Infectious Diseases, University of Alabama, Birmingham, Alabama, USA.; 5 Division of Infectious Diseases, The Ohio State University, Columbus, Ohio, USA.; 6 Division of Infectious Diseases, David Geffen School of Medicine, UCLA, Los Angeles, California, USA.; 7 Division of Infectious Diseases, Washington University, St. Louis, Missouri, USA.; 8 School of Medicine, University of Puerto Rico, San Juan, Puerto Rico.; 9 Division of AIDS, National Institute of Allergy and Infectious Diseases, Rockville, Maryland, USA.; 10 AIDS Network Coordinating Center, DLH Corporation, Bethesda, Maryland, USA.; 11 Clinical Monitoring Research Program Directorate, Frederick National Laboratory for Cancer Research, Frederick, Maryland, USA.; 12 Biostatistics Research Branch, Division of Clinical Research, National Institute of Allergy and Infectious Diseases, NIH, Bethesda, Maryland, USA.; 13 Department of Microbiology and Immunology, Medical University of South Carolina, Charleston, South Carolina, USA.; 14 School of Medicine and Skaggs School of Pharmacy and Pharmaceutical Sciences, UCSD, La Jolla, California, USA.; 15 Department of Medicine, University of Pennsylvania, Philadelphia, Pennsylvania, USA.; 16 The VRC 607/A5378 Study Team is detailed in Supplemental Acknowledgments.

**Keywords:** AIDS/HIV, Clinical trials, Drug therapy

## Abstract

**BACKGROUND:**

HIV-1–specific broadly neutralizing monoclonal antibodies (bNAbs) have emerged as promising interventions with the potential to effectively treat and prevent HIV-1 infections. We conducted a phase I clinical trial evaluating the potent CD4-binding site–specific (CD4bs-specific) bNAbs VRC01LS and VRC07-523LS in people with HIV-1 (PWH) not receiving antiretroviral therapy (ART).

**METHODS:**

Participants received a single intravenous 40 mg/kg dose of either VRC01LS (*n* = 7) or VRC07-523LS (*n* = 9) and did not initiate ART for a minimum of 14 days. The primary study objective was to evaluate safety and tolerability; the secondary study objectives were to evaluate pharmacokinetics (PK) and the impact of administered bNAbs on viral loads (VL) and CD4^+^ T cell counts in the absence of ART.

**RESULTS:**

This trial enrolled 16 PWH aged 20 to 57 years. Both bNAbs were safe and well tolerated. Mild local reactogenicity was only reported in participants who received VRC07-523LS, while both bNAbs were associated with mild systemic symptoms. Maximum serum concentrations (C_max_) following VRC01LS or VRC07-523LS were 1,566 ± 316 and 1,295 ± 376 μg/mL, respectively. VRC07-523LS administration significantly decreased VL in 8 out of 9 participants, with an average decline of 1.7 ± 0.8 log_10_ copies/mL within 14 days after administration. In contrast, VRC01LS administration resulted in a smaller average decline (0.8 ± 0.8 log_10_ copies/mL), and 3 out of 7 participants showedno change in VL. Postinfusion maximum decline in VL correlated with post hoc baseline in vitro viral susceptibility results for both bNAbs.

**CONCLUSION:**

The results of this trial support inclusion of potent CD4bs-specific bNAbs, such as VRC07-523LS, into next-generation treatment regimens for HIV-1.

**TRIAL REGISTRATION:**

ClinicalTrials.gov NCT02840474.

**FUNDING:**

National Institute of Allergy and Infectious Diseases (NIAID)/NIH (grants UM1 AI068634, UM1 AI068636, UM1 AI106701, UM1AI069424, UM1AI069501, UM1AI69415, UM1AI069534, UM1AI69494); the Intramural Research Program of the NIAID/NIH; National Center for Advancing Translational Sciences/NIH (grants UM1TR004548, UL1TR001881, and UL1TR001878); and the National Cancer Institute/NIH (contract 75N91019D00024).

## Abstract 2

Intravenously administered VRC01LS and VRC07-523LS were safe and demonstrated the capacity to reduce plasma viral loads in people with HIV-1 prior to antiretroviral therapy treatment.

## Introduction

HIV-1–specific broadly neutralizing monoclonal antibodies (bNAbs) have emerged as a promising therapeutic and prophylactic modality for HIV-1 (1). Developed in a subset of HIV-1 chronically infected individuals, bNAbs possess an exceptional ability to neutralize a wide range of heterologous HIV-1 strains. Notably, these bNAbs do not control viral replication in the donors from whom they were isolated, primarily due to the development of resistance (2). The majority of isolated bNAbs target semiconserved regions of HIV-1 envelope (ENV) protein, including the CD4-binding site (CD4bs), V1V2 loops of gp120, V3 loop, and the membrane-proximal external region (MPER) within the transmembrane region of gp41 (3). Unique features of bNAbs such as high levels of somatic hypermutation, long heavy chain complementarity determining regions 3 (HCDR3s), and poly- or autoreactivity to non–HIV-1 antigens in part explain the difficulty of inducing these neutralizing antibodies by vaccination (4). Recently, considerable attention has been directed toward the engineering and testing of second-generation bNAbs with improved neutralizing potential, increased breadth and potency, and extended serum half-lives (*t*_1/2_) (5, 6). Clinical evaluations have assessed the safety, pharmacokinetics (PK), and efficacy of multiple HIV-1 bNAbs administered alone or in combination in people with (PWH) or without (PWOH) HIV-1 and in HIV-exposed newborns. Overall, HIV-1 bNAbs are safe and well tolerated in these populations, with serum *t*_1/2_ ranging between 12 and 73.5 days (7–16). In PWH not suppressed on antiretroviral therapy (ART), bNAbs have demonstrated the ability to decrease the viral load (VL) of bNAb-sensitive viruses. However, the emergence of antibody-resistant viral variants and viral rebound after bNAb infusion is well documented (9, 10, 12, 14). Because bNAbs only effectively suppress sensitive viral strains, bNAb combinations more effectively limit rebound following analytical treatment interruption (11, 17–20). For sustained viral control and to address HIV-1 genetic diversity, it is probable that combinations of bNAbs targeting distinct regions of HIV-1 ENV will be required (21); however, optimal combinations are yet to be identified.

VRC01, a CD4bs-specific bNAb, was isolated from an individual with HIV-1 who managed the infection without ART for over 15 years (22). Two large, parallel, placebo-controlled VRC01 efficacy trials, known as the Antibody-Mediated Prevention (AMP) studies, provided proof of concept that bNAbs could be effective when used in a prophylactic setting. VRC01 was shown to prevent infection against VRC01-sensitive viruses, which corresponded to up to 30% of circulating viral strains. This resulted in an overall efficacy of up to 75% against the VRC01-sensitive isolates in these trials (23). These findings underscore the need for further optimization of individual HIV-1 bNAbs, and their combinations, to enhance the neutralization of real-world isolates and highlight the importance of evaluating HIV-1 bNAbs in relevant target populations.

After the discovery of VRC01, a clonal relative, VRC07-523, was engineered for greater viral neutralization breadth and potency. It was shown to be 5-fold more potent than VRC01, with a 50% inhibitory concentration (IC_50_) of less than 1 μg/mL against 92% of a 179 HIV-1 pseudovirus cross-subtype panel (21). LS mutations (ML428L and N434S) were engineered into the Fc region of both VRC01LS and VRC07-523LS to increase *t*_1/2_, and these modifications extended the VRC01 serum *t*_1/2_ from 15 to 71 days in PWOH (7). Furthermore, both VRC01LS and VRC07-523LS demonstrated favorable safety and PK profiles when administered to PWOH in phase I clinical testing (8). Given these promising clinical data from VRC01LS and VRC07-523LS in PWOH, we designed a multisite phase I clinical trial to evaluate safety, tolerability, PK, and the impact on VL and CD4^+^ T cell counts of a single intravenous (i.v.) administration of 40 mg/kg of either VRC01LS or VRC07-523LS in PWH not on ART.

## Results

**Figure 1:**
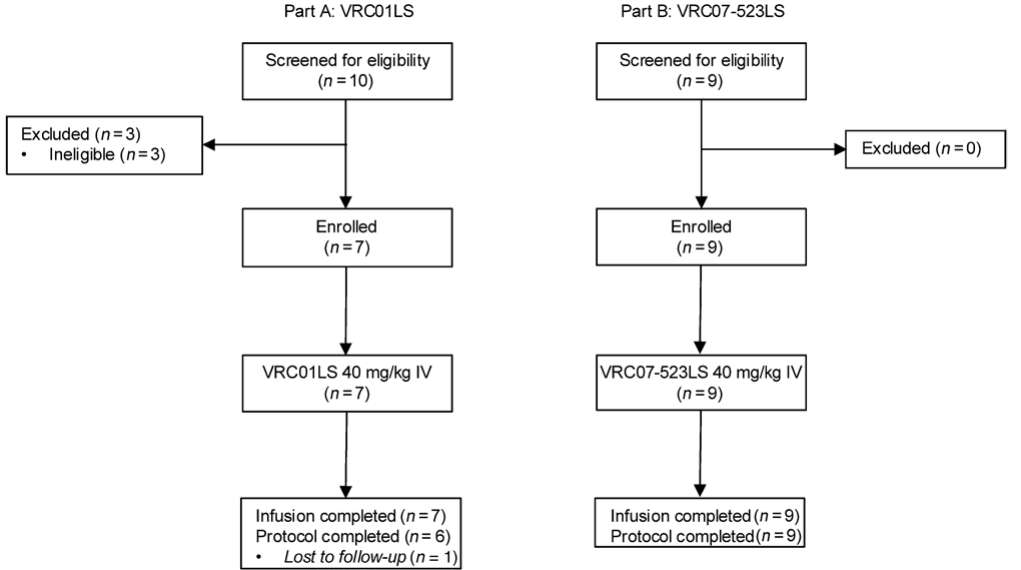
Study CONSORT diagram. Seven people with HIV-1 (PWH) were enrolled into Part A of the trial between April 2017 and October 2017 to receive VRC01LS, and 9 PWH were enrolled into Part B of the trial between November 2018 and January 2019 to receive VRC07-523LS. Study participants were not screened for susceptibility to VRC01LS or VRC07-523LS. Both study products were administered i.v. at a single dose of 40 mg/kg.

*Study population*. Of the 19 PWH screened for eligibility, 3 were ineligible to participate in this trial (Figure 1). Between April and October 2017, 7 PWH were enrolled into Part A of the trial and received 1 dose of VRC01LS at 40 mg/kg i.v. The demographic breakdown included 5 (71%) males and 2 (29%) females, with a median age of 39 (range 25 to 57) years. Two (29%) participants were White, and 5 (71%) were Black or African American (Supplemental Table 1; supplemental material available online with this article; https://doi.org/10.1172/jci.insight.181496DS1). The median baseline CD4^+^ T cell count was 523 (range 395 to 576) cells/μL and the median VL was 3,177 (range 404 to 173,134) copies/mL. Participants in Part A were not receiving ART at the time of enrollment into the study and only 1 of 7 had received prior ART treatment (Supplemental Table 2).

Between November 2018 and January 2019, 9 PWH were enrolled in Part B of the trial and received 1 dose of VRC07-523LS at 40 mg/kg i.v. This study cohort comprised 9 (100%) males with a median age of 26 (range 20 to 56) years. Seven (78%) participants were White, 1 (11%) was Black or African American male, and 1 (11%) was of undisclosed race. Two (22.2%) trial participants were Hispanic/Latino and 7 (77.8%) were Non-Hispanic/Latino (Supplemental Table 1). All 9 participants were ART naive at the time of enrollment. The median baseline CD4^+^ T cell count was 525 (range 382 to 827) cells/μL and the baseline VL was 37,987 (range 12,149 to 143,354) copies/mL (Supplemental Table 2). None of the study participants were prescreened for VRC01LS or VRC07-523LS sensitivity. Additional demographic and clinical characteristics of all participants can be found in Supplemental Tables 1 and 2.

**Figure 2:**
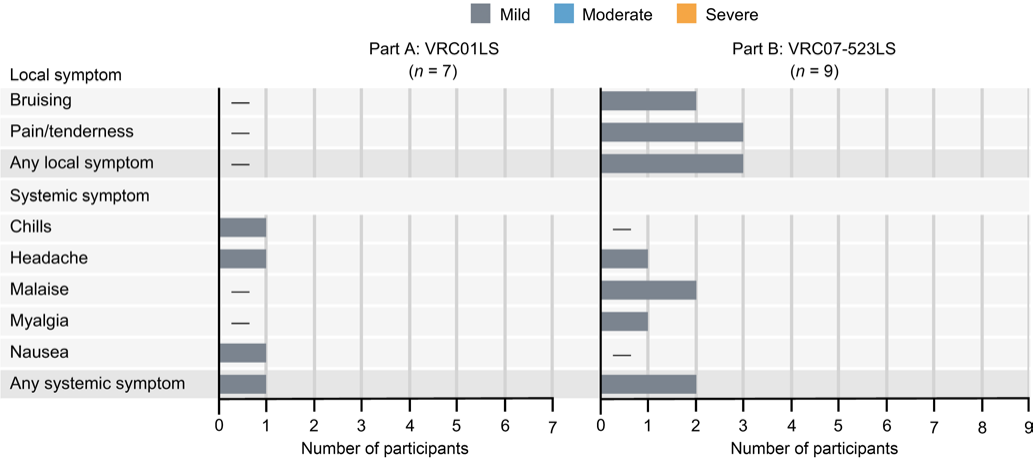
Reactogenicity followed VRC01LS and VRC07-523LS infusions. Number of participants (*x* axes) reporting solicited local or systemic symptoms (*y* axes) in the 3 days after infusion.

*Safety*. The primary objective of the trial was to evaluate safety and tolerability of VRC01LS and VRC07-523LS administered at 40 mg/kg i.v. to PWH. Both VRC01LS and VRC07-523LS demonstrated a favorable safety profile and were well tolerated (Figure 2 and Supplemental Table 3). Mild local reactogenicity was observed solely in participants who received VRC07-523LS and included bruising (*n* = 2 of 9, 22%) and pain/tenderness (*n* = 3 of 9, 33%) at the infusion site. Solicited systemic reactogenicity with both bNAbs was transient and mild. Following VRC01LS infusion, isolated cases of headache (*n* = 1 of 7, 14%), chills (*n* = 1 of 7,14%), and nausea (*n* = 1 of 7, 14%) were reported. Similarly, after VRC07-523LS infusion, single occurrences of headache (*n* = 1 of 9, 11.1%) or myalgia (*n* = 1 of 9, 11%) and 2 instances of malaise (*n* = 2 of 9, 22%) were reported. While no unsolicited adverse events (AEs) related to VRC01LS were reported, 2 unsolicited AEs related to VRC07-523LS administration were identified: one (*n* = 1 of 9, 11%) mild transient paresthesia that started in the last portion of the infusion and was resolved by the end of the infusion, and one (*n* = 1 of 9, 11%) severe transient neutrophil count decrease with the onset recorded on day 7 after VRC07-523LS administration (neutrophil count of 0.683 × 10^9^/L) and resolution without sequelae approximately 3 weeks later (neutrophil count of 2.708 × 10^9^/L). This transient decrease in neutrophil count was determined to be not clinically relevant and did not require further investigation. Neither VRC01LS nor VRC07-523LS resulted in any serious AEs (SAEs) and no antidrug antibodies (ADAs) were detected in the study participants following administration of either VRC01LS or VRC07-523LS (Supplemental Table 4).

**Figure 3:**
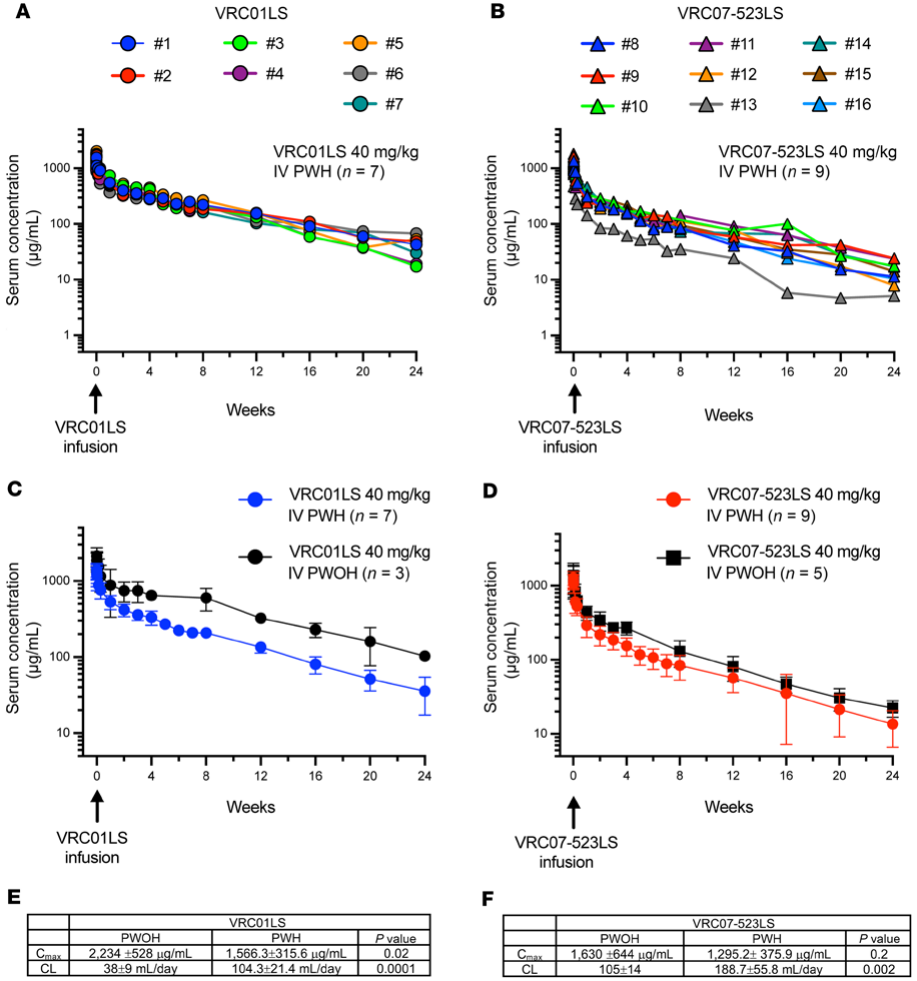
PK of VRC01LS and VRC07-523LS. Serum concentrations by individual study participants after a single 40 mg/kg i.v. infusion of VRC01LS (**A**) or VRC07-523LS (**B**). Geometric mean serum concentrations with error bars indicating SD of VRC01LS (**C**) and VRC07-523LS (**D**) administered at a single dose of 40 mg/kg i.v. to people with HIV-1 (PWH) as compared with previously published data in people without HIV-1 (PWOH) (7, 8). (**E** and **F**) Comparisons between C_max_ and CL in PWH vs. PWOH were performed using *t* test with unequal variance. C_max_, maximum serum concentration; CL, clearance.

*PK*. A secondary objective of the trial was to evaluate PK profiles of VRC01LS and VRC07-23LS. As such, we analyzed serum samples from all study participants up to 48 weeks after infusion. We observed persistent serum concentrations, with a gradual decline throughout the study period for both VRC01LS and VRC07-523LS (Figure 3, A and B, and Supplemental Figure 1, A and B). The estimated mean ± SD maximum serum concentration (C_max_) after a single i.v. infusion for VRC01LS was 1,566 ± 316 μg/mL, while a single i.v. infusion of VRC07-523LS yielded a slightly lower C_max_ of 1,295 ± 376 μg/mL. Two weeks (day 14) after bNAb administration and prior to ART in any participants, VRC07-523LS serum concentrations were significantly lower (*P* = 0.0001) as compared with VRC01LS at 231 ± 65 μg/mL and 423 ± 76 μg/mL, respectively (Supplemental Table 5). Evaluation of clearance (CL ± SD) and volume of distribution (V_d_ ± SD) also suggested differences between the antibodies. VRC07-523LS exhibited a more rapid CL at a rate of 188.7 ± 55.8 mL/day (*P* = 0.002) and a larger V_d_ of 15.6 ± 6.5 L (*P* = 0.006) compared with VRC01LS, which had a CL of 104.3 ± 21.4 mL/day and V_d_ of 7.1 ± 1.7 L. Finally, VRC01LS and VRC07-23LS displayed similar *t*_1/2_ ± SD of 47.3 ± 8.4 and 56.5 ± 13.2 days, respectively (Supplemental Table 5).

We conducted a post hoc comparative analysis to discern the differences between PK profiles of VRC01LS and VRC07-523LS in PWH with viremia and previously published PK profiles in PWOH (7, 8) (Figure 3, C and D). VRC01LS C_max_ was significantly lower in PWH as compared with PWOH when administered at the same dose of 40 mg/kg i.v. (*P* = 0.02), while VRC07-523LS C_max_ were similar between the 2 populations (Figure 3, E and F). Both bNAbs, however, displayed increased CL in PWH as compared with PWOH (VRC01LS *P* = 0.0001, VRC07-523LS *P* = 0.0018) (Figure 3, E and F).

*IgG1 allotypes*. We investigated the potential impact of IgG1 genetic markers (GMs), known as allotypes, on the PK parameters of bNAbs (24). IgG1 allotypes of each study participant were assessed (Supplemental Table 2). In the VRC01LS arm of the trial, the majority (*n* = 5 of 7, 71%) of participants were homozygous for the GM17/17 allotype, while 2 (*n* = 2 of 7, 29%) participants were heterozygous for the GM3/17 allotypes. In contrast, the majority (*n* = 7 of 9, 78%) of participants in the VRC07-523LS arm of the trial were heterozygous for the GM3/17 allotypes, and 2 (*n* = 2 of 9, 22%) participants were homozygous for the GM17/17 allotype. However, the presence of these allotypes did not markedly impact the PK profiles of VRC01LS or VRC07-523LS, which contain the GM3 and GM17,1 allotypes, respectively.

*Postinfusion functionality of administered bNAbs*. To confirm the functionality of the administered bNAbs after infusion, we performed neutralization assays to evaluate participant serum in a small panel of HIV-1 pseudoviruses with a known range of sensitivities to VRC01LS and VRC07-523LS. We found both VRC01LS and VRC07-523LS retained neutralizing activity following administration and as expected, showed a range of sensitivities to the viruses in the testing panel (Supplemental Table 6).

*Antiviral activity*. Another secondary objective of the trial was to evaluate the antiviral activity of VRC01LS and VRC07-523LS in the absence of ART. At the time of VRC01LS or VRC07-523LS infusions, all study participants were ART naive except for 1 participant in the VRC01LS arm of the trial who was on an ART holiday (Supplemental Table 2). All study participants were closely monitored for signs of HIV-1 progression after bNAb infusion for the duration of the study. In line with standard of care (SOC) guidelines, initiation of ART was strongly encouraged for all participants 4 weeks after VRC01LS infusion and 14 days following administration of VRC07-523LS. Most participants followed this recommendation; however, delays in the receipt of the results of the HIV-1 VL testing and subsequent scheduled visits resulted in occasional delays in ART initiation. In the VRC01LS arm, 3 participants (1, 2, and 3) who maintained relatively low VLs of less than 7,000 copies/mL and stable CD4^+^ T cell counts chose to delay ART initiation (Supplemental Figure 1A and Supplemental Table 2). Participant 4 did not report ART initiation, but tested positive for ART (EVG, FTC, and TFV) 24 weeks after VRC01LS infusion. Three participants (5, 6, and 7) with relatively high baseline VL and/or little to no VL decrease after VRC01LS administration started ART 5 to 12 weeks after infusion. In the VRC07-523LS arm, 5 study participants (11, 12, 14, 15, and 16) initiated ART 2 weeks after VRC07-523LS infusion; the remaining 4 study participants (8, 9, 10, and 13) started ART 3 to 5 weeks after infusion (Supplemental Figure 1B and Supplemental Table 2). Time points that occurred after ART initiation were not considered in the analyses conducted to assess the impact of bNAbs on HIV-1 VL and CD4^+^ T cell counts.

**Figure 4:**
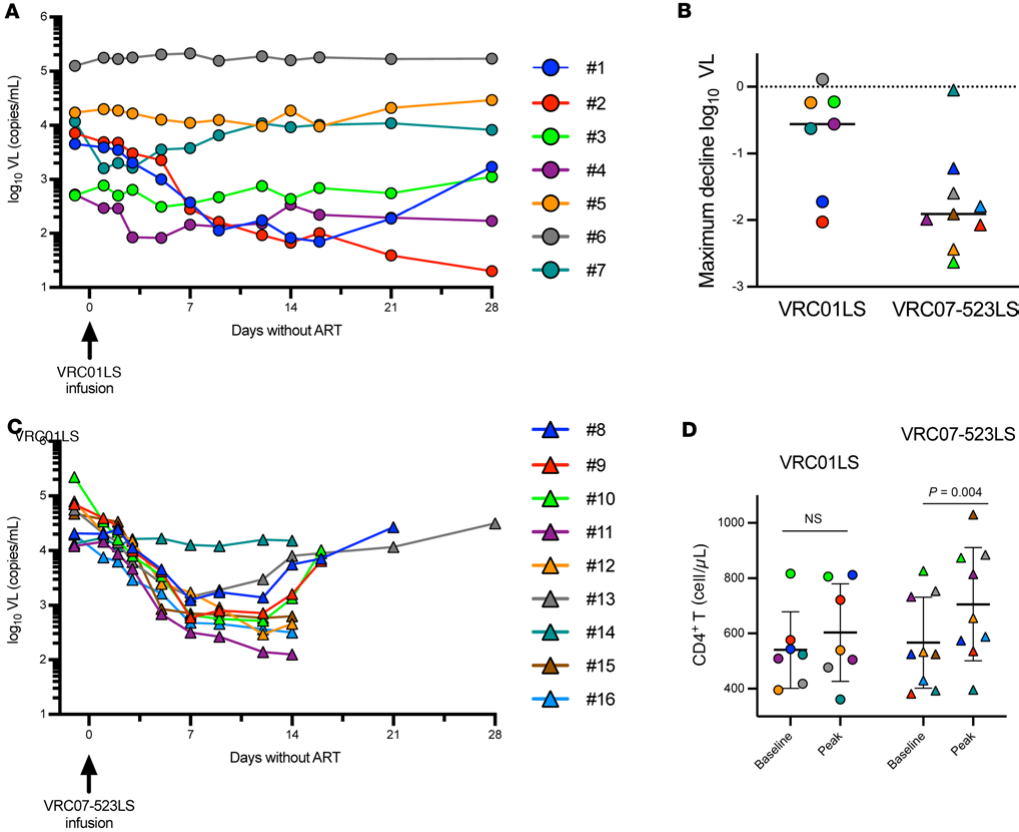
The effect of VRC01LS and VRC07-523LS on HIV-1 viral load and CD4^+^ T cell counts after infusion. Plasma viral load (VL) after a single 40 mg/kg i.v. infusion of VRC01LS (**A**) and VRC07-523LS (**B**) for the first 28 days prior to initiation of antiretroviral therapy (ART). (**C**) Maximum VL decline in participants infused with VRC01LS or VRC07-523LS prior to ART initiation (by day 14); *P* = 0.055 based on Wilcoxon’s rank-sum test. Solid horizontal lines indicate group median. (**D**) Changes in CD4^+^ T cell counts after a single 40 mg/kg i.v. infusion of VRC01LS and VRC07-523LS from baseline to peak CD4^+^ T cell counts by day 14 prior to ART. Wilcoxon’s rank-sum test was used for comparisons; lines indicate group means with SD; *P* value was calculated based on Wilcoxon’s signed-rank test.

Following a single 40 mg/kg i.v. infusion of VRC01LS, we observed pronounced reductions in VL in 2 of 7 participants: a 1.7 log_10_ copies/mL VL decline (participant 1) and a 2.0 log_10_ copies/mL VL decline (participant 2) by day 14 (Figure 4A and Supplemental Table 7). However, after the initial decline, VL rebounded and nearly reached baseline levels by day 21 and 84 (participants 1 and 2, respectively) (Supplemental Figure 1). At least a 0.5 log_10_ copies/mL decline in VL was observed in 2 participants (4 and 7) 5 to 7 days after infusion, but not in 3 other participants (3, 5, and 6), suggesting the predominant circulating virus in these participants was relatively resistant to VRC01LS. Overall, the mean ± SD decline in VL was 0.8 ± 0.8 log_10_ copies/mL and the time to reach a minimum VL ranged from 1 day to 28 days after infusion (Figure 4A and Supplemental Table 7).

In contrast, 8 out of 9 study participants displayed pronounced declines in VL following a single 40 mg/kg i.v. administration of VRC07-523LS (Figure 4B and Supplemental Table 7). The VL of 3 participants (9, 10, and 12) decreased over 2 log_10_ (range 2.1 to 2.6) copies/mL, while those of the other 5 (8, 11, 13, 15, and 16) decreased from 1.2 to 2.0 log_10_ copies/mL. Only 1 study participant (14) had no change in VL from baseline. The mean ± SD decline in VL in all 9 participants was 1.7 ± 0.8 log_10_ copies/mL following administration of VRC07-523LS. Overall, time to reach minimum VL ranged from 2 to 14 days (Figure 4B and Supplemental Table 7), at which point participants in this arm were encouraged to initiate ART. A viral rebound of at least a 0.5 log_10_ copies/mL increase from minimal VL was observed in the 4 study participants (8, 9, 10, and 13) who did not initiate ART on day 14 (ranging from 16 to 21 days after infusion). No participant’s VL decreased to undetectable levels in either arm.

In our exploratory analyses, we examined the ability of each bNAb to induce VL decline. We compared the peak reduction in VL from baseline between participants who received either VRC01LS or VRC07-523LS. Since this trial was not designed to directly compare these 2 bNAbs, our assessment was limited to the first 14 days after infusion, before any participant in the trial initiated ART. This comparison in maximum decline in VL trended toward statistical significance (*P* = 0.055), with VRC07-523LS infusion leading to a more pronounced decline (Figure 4C).

Changes in CD4^+^ T cell counts. The final secondary trial objective was to assess the impact of a single VRC01LS or VRC07-523LS administration on CD4^+^ T cell counts in the absence of ART. To this end, we compared baseline CD4^+^ T cells to peak CD4^+^ T cells after baseline, but within 14 days after administration. The peak increase in CD4^+^ T cell counts occurred on average 12 days after VRC01LS and 10 days after VRC07-523LS administration (Supplemental Table 8). A modest uptrend in CD4^+^ T cell counts was observed in participants who received VRC01LS, with a median increase of 59 cells/μL, and a statistically significant increase in CD4^+^ T cells (*P* = 0.004) was observed in participants who received VRC07-523LS, with a median increase of 82 cells/μL (Figure 4D and Supplemental Table 8).

**Figure 5:**
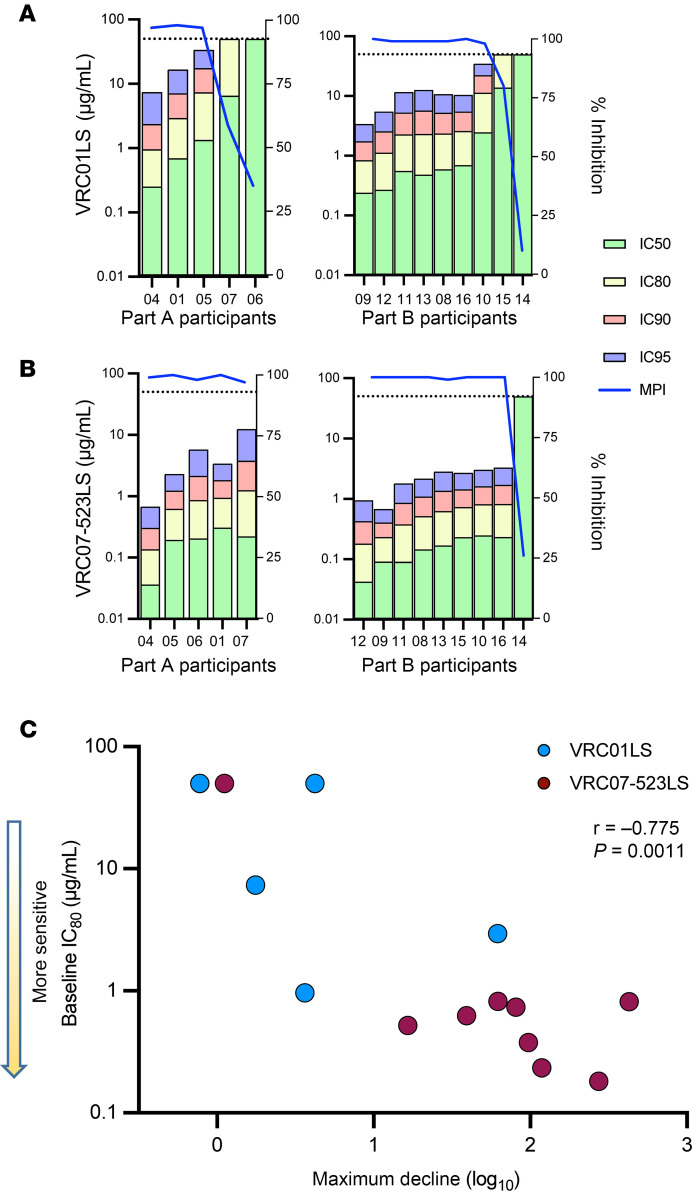
Baseline sensitivity of participants’ viruses to VRC01LS and VRC07-523LS. Preinfusion baseline virus from all participants in the trial were tested for sensitivity to VRC01LS (**A**) and VRC07-523LS (**B**) using the Monogram PhenoSense assay. Individual inhibitory concentrations for 50%, 80%, 90%, and 95% neutralization were inferred and indicated with different colors and graphed from most sensitive participant virus to least sensitive for each antibody. The results are graphed by which part of the trial the participants were enrolled (Part A participants went on to be infused with VRC01LS and Part B participants went on to be infused with VRC07-523LS). (**C**) Maximum viral load (VL) decline by day 14 in participants is associated with baseline sensitivity of the virus to the infused antibody. Each dot represents a single participant and is filled with color to indicate which antibody was infused. MPI, maximum percentage inhibition. Spearman’s rank correlation test was used to derive *r* and *P* values.

*Viral resistance*. To gain deeper insights into the viral decline after a single infusion of VRC01LS or VRC07-523LS, we measured the neutralization sensitivity of the participants’ baseline viruses using the Monogram PhenoSense monoclonal antibody (mAb) assay (Figure 5, A and B, and Supplemental Table 9). The baseline viruses for 2 participants who were enrolled into the VRC01LS arm of the trial could not be amplified, but of the remaining 14 participants, 13 had baseline virus that displayed some level of sensitivity to at least 1 bNAb, while 1 participant (participant 14) harbored virus resistant to both antibodies (Figure 5A). When defining neutralization sensitivity as an IC_80_ of less than 1 μg/mL, baseline viruses from only 2 of 14 participants were sensitive to VRC01LS (Figure 5A), while 12 were sensitive to VRC07-523LS (Figure 5B), highlighting the increased potency of VRC07-523LS compared with VRC01LS. Overall, there was a significant correlation between increased neutralization sensitivity at baseline and a more pronounced peak decline in VL after infusion (*r* = 0.775; *P* = 0.001) (Figure 5C).

To account for the concentration of the infused antibody, we calculated the predicted serum neutralization 80% inhibitory dilution titer (PT_80_) value, which is defined as a biomarker to quantify the neutralization potency of antibodies in an individual’s serum against an HIV-1 isolate (25). In the context of this trial, we utilized the measured C_max_ of infused bNAb and the Monogram PhenoSense–defined baseline IC_80_. At the infusion time point, the PT_80_ biomarker correlated with maximum decline in VL within the first 14 days more significantly than IC_80_ alone (Spearman’s rank correlation *r* = –0.82; *P* = 0.0002) (Supplemental Figure 1C).

**Figure 6:**
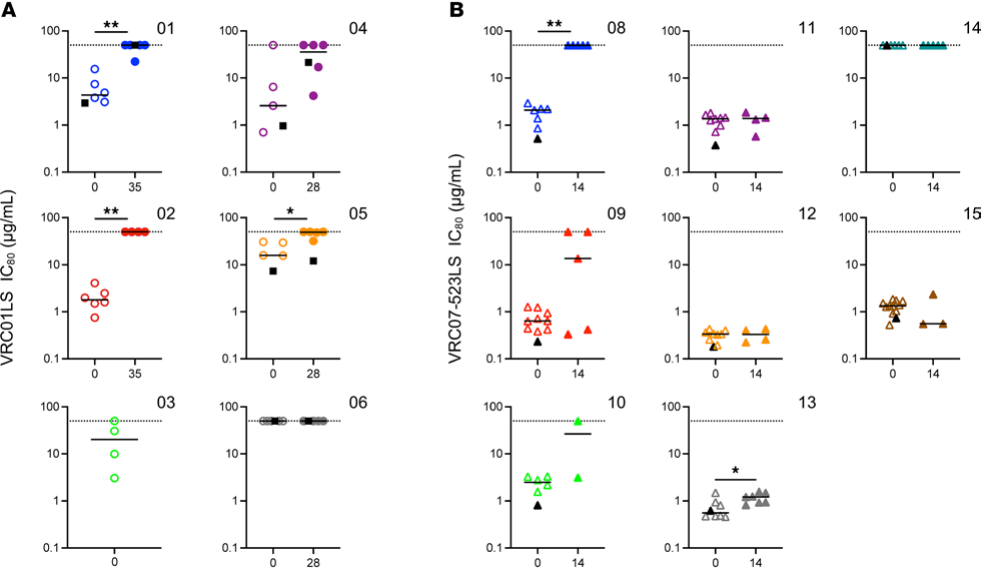
Baseline sensitivity of participants’ viruses to VRC01LS and VRC07-523LS. ENVs cloned from baseline and 1 month after infusion for each participant were tested for neutralization sensitivity to the infused antibody VRC01LS (**A**) or VRC07-523LS (**B**). IC_80_ values of each ENV clone are plotted for 2 time points (before infusion is open symbol and after infusion is filled symbol). ENVs could not be amplified from postinfusion participant 3. Black line indicates geometric mean IC_80_. Pre- and postsensitivity was compared by Mann-Whitney *U* test. *P < 0.05, **P < 0.01. Black-filled symbols indicate the IC_80_ calculated by the Monogram PhenoSense assay, which could not be calculated for participants 2 and 3. The post time point was only measured at the matched time point for participants 1, 4, 5, and 6.

To test the effect of the infused antibody on each participant’s virus population, we generated single genome ENV sequences and cloned 4 to 6 of them from before and after the bNAb infusion. Pseudoviruses expressing these clones were then assessed for their sensitivity to the infused antibodies by standard TZM-bl neutralization assay. Two other HIV-1 bNAbs were included for reference values: 3BNC117, a CD4bs HIV-1 bNAb, and 10E8, a gp41 MPER-targeting HIV-1 bNAb (Figure 6 and Supplemental Table 10). In the VRC01LS arm, ENVs from 3 of the 6 participants (1, 2, and 5) tested were characterized by significantly increased viral resistance to VRC01LS after infusion (Figure 6A). It was not possible to amplify viral env sequences from participant 7 nor postinfusion virus from participant 3, so no comparisons could be performed. All participant 6 ENVs were completely resistant at baseline and remained resistant after infusion. Participant 4 had the greatest variation in baseline virus phenotype and harbored 1 preexisting resistant ENV out of 4 tested. After infusion, the ENVs trended toward significantly different (*P* = 0.09) from preinfusion, and 3 out of 5 ENVs were resistant to the infused bNAb. In the VRC07-523LS arm, it was not possible to amplify viral env sequences for participant 16, so no further analyses were performed, and ENVs from participant 14 were completely resistant to the bNAb at baseline and after infusion (Figure 6B and Supplemental Table 11). For the remaining 7 participants, 2 had significantly increased virus resistance to the infused antibody 14 days after infusion (8 and 13). Participants 9 and 10 did have detectable resistant ENVs after infusion (3 out of 5 ENVs for participant 9 had IC_80_s > 10 μg/mL and 1 out of 2 ENVs for participant 10 was resistant). For 3 participants (11, 12, and 15) no difference in ENV sensitivity was measured between baseline and after infusion, aligning with the observation that VLs in these participants were still declining and had not yet started to rebound by day 14 (Supplemental Table 7). Overall, these data demonstrate that under monotherapy, when the virus is not fully suppressed, resistance to the infused antibody develops.

### Discussion

In this phase I clinical trial, we evaluated the safety, tolerability, PK, and impact on VL and CD4^+^ T cell counts of a single dose of 2 CD4bs bNAbs, VRC01LS and VRC07-523LS, in PWH with CD4^+^ T cell counts of 350 cells/μL or greater who were not on ART during the study screening, enrollment, or bNAb infusion. Both bNAbs had favorable safety profiles when administered i.v. at 40 mg/kg. Mild local reactogenicity was recorded only for VRC07-523LS. Systemic reactogenicity was mild and short-lived for both bNAbs, and no ADA was detected following bNAb infusions.

VRC01LS and VRC07-523LS exemplify next-generation bNAbs with enhanced PK profiles, as compared with the initially isolated bNAb VRC01. Prolonged serum *t*_1/2_ values have been reported in PWOH for both bNAbs (7, 8) and in this trial, we extend these findings to viremic PWH not on ART. In this trial, the estimated *t*_1/2_ of VRC01LS and VRC07-523LS in PWH were 47 and 57 days, respectively. We also report differences in PK profiles between the two bNAbs. Overall, VRC07-523LS exhibited faster CL, larger V_d_, and marginally lower C_max_ as compared with VRC01LS. Furthermore, VRC07-523LS demonstrated increased in vitro activity (lower IC_80_) and higher PT_80_. Likewise, VL reduction was more pronounced in participants who received VRC07-523LS, demonstrating the antibody’s superior neutralization breadth and potency was retained following administration. Post hoc analysis also revealed differences in the PK parameters of VRC01LS and VRC07-523LS between PWOH and PWH populations. Specifically, PWH exhibited lower peak serum concentrations and faster CL rates. More rapid rates of elimination in PWH have been reported for other HIV-1 bNAbs and are possibly linked to viral binding of bNAbs and changes in antibody metabolism influenced by chronic inflammation, among other contributing factors (9, 10, 26). Reported *t*_1/2_ values in PWH and PWOH were within a similar range; however, larger studies are needed to further investigate these findings. There are some data to suggest that IgG1 GM allotype impacts the PK profile of an administered antibody (24, 27). For example, when the G1m17,1 mAb infliximab was given to G1m17,1 homozygous patients, it cleared faster and had a shorter *t*_1/2_ as compared with heterozygous G1m3,-1 patients. This effect was attributed to differential binding affinities of IgG1 allotypes to the FcRn receptor (24). To gain additional insight about the PK profiles of VRC01LS and VRC07-523LS, we examined the potential impact of patient IgG1 GM allotypes on PK parameters. We found that IgG1 allotypes did not impact PK profiles of VRC01LS or VRC07-523LS regardless of whether the allotypes of study participants and therapeutic bNAb were matched.

The increases observed in CD4^+^ T cell counts in response to HIV bNAb therapy in PWH have not been previously reported to our knowledge. The significance of these observed increases warrants further in-depth investigation to elucidate the underlying mechanisms driving such a rapid rise. Available data pertaining to early CD4^+^ T cell count increments after ART initiation remain limited (28–30) and the relatively small sample size of our study poses difficulties in making robust comparisons across different cohorts and drug classes. However, despite these limitations, the swift surge in CD4^+^ T cell counts observed following VRC07-523LS initiation and marginal increase in CD4^+^ T cell counts after VRC01LS suggest a potentially dynamic process involving the mobilization of immune cells from lymph nodes. Comprehensive studies focusing on the interplay between immune cell trafficking, cytokine dynamics, and viral reservoirs after the initiation of bNAb therapy will be essential for a comprehensive understanding of the observed CD4^+^ T cell count increases.

VRC01 and VRC07-523 are clonal relatives of each other and target the same epitope within the CD4bs on the viral ENV protein. However, preclinical data demonstrated that VRC07-523 is broader and more potent on large pseudovirus panels as measured by in vitro neutralization assay (21). This raised the question of whether VRC07-523’s in vitro potency and breadth would translate into enhanced in vivo efficacy in PWH. We found that VRC07-523LS neutralized participants’ preinfusion virus more potently than VRC01LS, as measured by Monogram’s PhenoSense bulk virus neutralization assay. This estimated preinfusion sensitivity of the virus (quantified as an IC_80_) correlated with the maximum decline in VL, suggesting that the PhenoSense mAb assay may have predictive value for antibody efficacy. In this trial, the more potent antibody, VRC07-523LS, decreased VL by a mean of 1.7 ± 0.8 log_10_ copies/mL, while VRC01LS decreased VL by 0.8 ± 0.8 log_10_ copies/mL 14 days after administration. It is important to note that no prescreening occurred for this clinical trial; therefore, multiple participants were enrolled who had preexisting resistance to the infused bNAbs.

Interestingly, the PT_80_ biomarker, which was originally designed to estimate bNAb prevention efficacy, may have utility in the context of therapy. We observed a strong correlation between PT_80_ on day of infusion and maximum decline in VL within the first 14 days after infusion (Supplemental Figure 1C). Post hoc analysis of the AMP trial suggested that a PT_80_ of greater than 200 would lead to 90% efficacy against HIV-1 acquisition (25); however, threshold values in the context of the treatment are yet to be established. Here, we report PT_80_ greater than 500 for all participants with at least 1 log_10_ VL reduction. Additional studies are necessary to understand the relationship between in vitro neutralization assays and biomarkers such as PT_80_.

The question of the necessity of technology for prescreening participants’ virus for bNAb sensitivity before being enrolled in a clinical trial is currently an active area of research (31). Post hoc analyses of the neutralization potency of bNAbs against individual ENV clones obtained from each participant highlighted heterogeneity in virus sensitivity to these antibodies. In general, when comparing the Monogram PhenoSense assay for baseline screening to the single-ENV cloning approach, the IC_80_ values of the PhenoSense mAb assay closely matched the geometric mean IC_80_ from the individually cloned ENVs (Figure 6). However, it is important to note that participant 4 harbored baseline virus that was phenotypically heterogeneous by single-ENV cloning, with IC_80_ values ranging from 0.70 to 50 μg/mL in the 4 ENVs tested. The PhenoSense assay measured a baseline IC_50_ of 0.25 μg/mL, which falls within a common eligibility criteria of IC_50_ less than or equal to 0.25 μg/mL (31, 32), suggesting that resistant variants occurring at low frequency may be undetected in this assay.

This trial has several limitations. While the limited number of participants is consistent with the typical scope of phase I trials, such a small cohort diminishes the ability to extrapolate these findings to a broader population. Furthermore, due to SOC requirements, most of the study participants initiated ART during the follow-up period. This limited any long-term assessment of the impact of VRC01LS and VRC07-523LS on VLs and CD4^+^ T cell counts. Lastly, we were unable to amplify pre- and postinfusion virus clones of several participants, and therefore could not determine virus sensitivity to the infused antibody for all participants. It is important to note that the gap between the enrollment into 2 arms of the study was due to the sequential development and availability of VRC01LS and VRC07-523LS. Furthermore, the 2 arms of this study were not designed for comparison and had different enrollment and ART treatment initiation criteria. Post hoc analyses of the results are, therefore, meant to be hypothesis generating.

Collectively, these data demonstrating potent antiviral activity in viremic PWH, rapid increases in CD4^+^ T cell counts, and favorable PK profiles, support further development of next-generation bNAbs against HIV-1. bNAb combinations optimized to sustain target PT_80_ values could be used together with long-lasting ARTs for both treatment and prevention. Additionally, bNAbs continue to be evaluated in the absence of ART in prevention settings, such as long-acting pre-exposure prophylaxis and prevention of vertical transmission of HIV-1. Since the initiation of this trial, a series of studies have been either completed or are currently ongoing involving VRC01LS or VRC07-523LS. These evaluations include a diverse range of single- and multiple-dose protocols, combinations with other bNAbs, and alternative pharmaceutical agents in both pediatric and adult populations (e.g., ClinicalTrials.gov NCT02256631, NCT03721410, NCT03735849, NCT03803605, NCT05281510, NCT03928821, NCT03205917, and NCT04983030). Such studies promise to enhance our understanding of VRC01LS and VRC07-523LS’s effectiveness in PWH, paving the way for enhanced tools in the prevention and treatment of HIV-1.

## Methods

### Sex as a biological variable.

Male and female participants were enrolled in this study, which was open to all sexes. Randomization included matching for sex.

### Study design and population.

This was a phase I, open-label, single-dose clinical trial (NCT02840474) examining the safety, PK, and virologic effects of the HIV-1 specific bNAbs VRC01LS (Part A) or VRC07-523LS (Part B) administered i.v. to viremic PWH. This multicenter clinical trial was sponsored and conducted by the Vaccine Research Center (VRC), National Institute of Allergy and Infectious Diseases (NIAID), NIH in collaboration with AIDS Clinical Trials Group (ACTG Network). Part A of the trial was conducted at the University of Pennsylvania Clinical Research site (CRS), University of Pennsylvania (UPenn), Philadelphia, Pennsylvania, and Part B of the trial was conducted at 8 ACTG-designated sites that included UPenn; UCLA CRS Center for Clinical AIDS Research and Education (CARE), Los Angeles, California; Alabama CRS, University of Alabama (UAB), Birmingham, Alabama; Washington University Therapeutics (WT) CRS, St. Louis, Missouri; Cincinnati CRS, University of Cincinnati, Cincinnati, Ohio; Puerto Rico AIDS Clinical Trials Unit CRS, San Juan, Puerto Rico; and Ohio State University CRS, Columbus, Ohio. The Investigational New Drug (IND) was sponsored by the Division of AIDS (DAIDS), NIAID.

Eligible study participants were PWH, clinically stable adults 18–70 years of age with at least one tested plasma VL of 500 copies/mL or greater and CD4^+^ T cell count of 350 cells/μL or greater (on 2 out of 3 consecutive testings) within 28 days of enrollment. Exclusion criteria were previous receipt of a humanized or human mAb, and changes in ART regimen in the 12 months prior to enrollment whether on or off ART (Part A) or prior use of ART (Part B). A full list of inclusion and exclusion criteria is included in the trial protocol.

### Product and procedures.

VRC01LS and VRC07-523LS were produced under current Good Manufacturing Practice (cGMP) by VRC, NIAID, NIH at the VRC Vaccine Pilot Plant (VPP) operated under contract by the Vaccine Clinical Materials Program (VCMP), Leidos Biomedical Research, Inc. VRC01LS and VRC07-523LS are human bNAbs targeted against the HIV-1 CD4bs. VRC01LS was engineered by site-directed mutagenesis of VRC01 and VRC07-523LS was engineered by pairing the VRC01 light chain with the VRC07 heavy chain, which was identified by the next-generation sequencing of antibody gene transcripts in the VRC01 donor, as previously described (5, 33). LS mutation was added to increase binding affinity to the neonatal Fc receptor and extend plasma *t*_1/2_ (5).

VRC01LS or VRC07-523LS was administered i.v. as a single dose of 40 mg/kg. The dosages in this trial were determined based on previous studies of VRC01LS and VRC07-523LS in healthy adults (7, 8). All product administrations were monitored by a study clinician and study participants were observed for at least 30 minutes following product administration. Reactogenicity symptoms were solicited daily for 3 days after product administration and unsolicited AEs were recorded through 56 days following study product administration.

All study participants continued follow-up with their primary HIV care provider for the duration of the study. Progression of HIV was monitored at protocol-defined intervals by testing CD4^+^ T cell counts and HIV-1 RNA PCR VL; a decrease in CD4^+^ T cell counts to less than 200 cells/μL would have prompted referral for clinical evaluation and consideration of fully suppressive ART initiation. In addition, study participants in Part A were encouraged to initiate ART if there was a 30% drop in CD4^+^ T cell counts or in the event of any increase in VL at the discretion of the primary HIV care provider. Study participants in Part B were encouraged to initiate 3-drug ART as prescribed by their primary HIV clinician any time after completing day 14 of study evaluations; the differences in ART initiation in Part A and Part B of the trial were prompted by the ongoing evolution of the SOC guidelines. All participants, regardless of ART status, were followed for safety purposes according to the study schedule of evaluations.

### PK analysis.

Quantification of VRC01LS or VRC07-523LS concentrations in participant serum was performed as previously described (7, 8). Briefly, PK enzyme-linked immunosorbent assay (ELISA) was performed by utilizing a Beckman Coulter Biomek FX automated liquid handler, operated through Beckman Coulter SAMI EX software. Plates coated with 5C9 MAB anti-VRC0X capture antibody (34) were incubated overnight, blocked, and then detected with mouse anti-human IgG1 horseradish peroxidase–conjugated antibody (Invitrogen, S-1599) followed by brief incubation with tetramethylbenzidine (TMB) substrate. A Molecular Devices Paradigm multi-mode reader was utilized to measure optical density at 450 nm. Sample concentrations were determined using a standard curve consisting of 8 standard concentrations covering the range of 0.1 μg/mL to 0.005 μg/mL and fitted by linear regression analysis.

PK analysis was performed using both noncompartmental methods for VRC01LS and VRC07-523LS on samples collected prior and up to 48 weeks after the infusion; Phoenix 8.0 (Certara) was used to perform this analysis. Calculated PK parameters included area under the curve (AUC), C_max_, CL, V_d_, terminal elimination rate constant (λ_z_), and the terminal *t*_1/2_. C_max_ was taken directly from the observed concentration-time data. The terminal slope, λ_z_, was be determined from the log-linear portion of the curve and the terminal *t*_1/2_ calculated as 0.693/λ_z_. AUC_0_-C_last_ was determined using the linear trapezoidal method, where C_last_ is the concentration at week 48 or earlier if earlier samples were below the quantitative limit. If the final sample, C_last_, had measurable VRC01LS or VRC07-523LS concentrations, the remaining AUC after the final concentration (AUCC_last-inf_) was estimated as C_last_/λ_z_. CL was calculated as dose/AUC_0-inf_ and V_z_ as CL/l_z_.

### PhenoSense mAb assay.

A PhenoSense mAb assay was used to assess the susceptibility of pseudovirions incorporating HIV-1 ENV proteins to VRC01LS and VRC07-523LS in all study participants. This assay was developed and performed by LabCorp-Monogram Biosciences. Briefly, populations of full-length ENV sequences were amplified from plasma-derived HIV RNA from study participants and cloned into an ENV expression vector to create populations with diverse ENV sequences. These plasmids were cotransfected into the cells with an HIV genomic reporter containing luciferase instead of the *env* gene to generate luciferase reporter pseudovirions capable of a single round of entry into cells. Pseudovirions were tested for neutralization sensitivity to serially diluted VRC01LS and VRC07-523LS using an in vitro cell-based assay. The concentration of VRC01LS or VRC07-523LS required to inhibit virus infectivity by specific percentages 50%, 80%, 90%, and 95% (IC_50_, IC_80_, IC_90_, and IC_95_, respectively) and maximum percentage inhibition (MPI) were assessed based on LabCorp-Monogram Biosciences protocols.

### Single-genome sequencing and cloning of gp160 env genes for Part A.

Plasma virus *env* genes were amplified and cloned as previously described, with the following modifications (12). Virions were concentrated from 0.3 mL to 1.8 mL of plasma by centrifugation, and the supernatant was removed. Viral RNA was extracted from 140 μL concentrated supernatant using the QIAamp Viral RNA Mini Kit (52906, Qiagen) according to the manufacturer’s instructions and were immediately reverse transcribed. A reverse transcription (RT) reaction with a total volume of 50 μL included 500 μM dNTP (10297018, Thermo Fisher Scientific), 100 nM gene-specific RT primer (envB3out, TTGCTACTTGTGATTGCTCCATGT), 100 units of SuperScript III reverse transcriptase (18080093, Thermo Fisher Scientific), 1× First-Strand Buffer, 1 mM DTT, 20 units of RNase inhibitor (10777019, Thermo Fisher Scientific), and template RNA or nuclease-free water. First, a mixture of RNA template, dNTP, and RT primer was incubated for 10 minutes at 65°C to denature the RNA, followed by incubation on ice (at least 2 minutes). The remaining reagents were added into the denatured RNA, followed by incubation for 50 minutes at 50°C and 10 minutes at 85°C. Quantification of the cDNA was conducted by limiting-dilution PCR using fluorescence-assisted clonal amplification (35) with a mixture of 2 forward primers (CCGGCTGGTTTTGCGATTCTAAAATG and CCGGCTGGTTTTGCGATTCTAAAGTG) and 4 reverse primers (ATGGGAGGGGCATACATTGCTTT, ATGGGAGGGGCATACATTGCTCT, ATGGGAGGGGCATACATTGCCTT, and ATGGGAGGGGCATACATTGCCCT).

Amplification of *env* genes was performed by nested PCR using Platinum Taq High Fidelity polymerase (Invitrogen), as previously described (36, 37). To ensure that a majority of amplicons would be generated from a single cDNA template, cDNA was diluted so that PCR-positive wells were fewer than 30% of the reactions. Single cDNA templates were amplified in a 35-cycle first-round PCR with forward and reverse primers envB5out (TAGAGCCCTGGAAGCATCCAGGAAG) and envB3out (TTGCTACTTGTGATTGCTCCATG), followed by a 45-cycle second-round PCR containing 1 μL of the first-round product and forward and reverse primers envB5in (caccTTAGGCATCTCCTATGGCAGGAAGAAG) and envB3in (GTCTCGAGATACTGCTCCCACCC). All PCR procedures were performed in PCR clean rooms free of post-PCR or plasmid DNA. Amplicons were run in 1% agarose gels and sequenced by ACGT Inc. Sequences that contained stop codons, large deletions, or mixed bases were removed from further analysis *env* genes from pre- and postinfusion time points were cloned by reamplifying 2 μL of first-round PCR template with 0.2 μL of 20 μM forward and reverse primers envB5in and envB3in (first round DNA from participant 5 was reamplified with envB5in and envB3out) in a total reaction volume of 20 μL using Phusion Hot Start Polymerase (New England Biolabs) according to the manufacturer’s instructions, with an annealing temperature of 64°C and an extension time of 1 minute for 26 cycles. Amplicons were cloned using the pcDNA3.1 Directional TOPO kit (Invitrogen) and transformed in XL2-Blue MRF′ Ultracompetent Cells (Agilent Technologies), all according to the manufacturers’ instructions. Transformants were plated on LB plus ampicillin (100 μg/mL) plates (K·D Medical) overnight at 37°C. Colonies were cultured, and DNA was isolated using the 24-well blocks and a QIAprep 96 Plus Miniprep Kit (Qiagen). All plasmids were sequence verified before expression. For 2 participants (2 and 6), because we were unable to clone the majority of the genes, we used the strategy of adding a CMV promoter and BGH polyadenylation site to the HIV amplicon through PCR, as previously described (38).

### High-throughput single-genome sequencing and synthesis of gp160 env genes for Part B.

For RNA extraction, cDNA synthesis and quantification in Part B, while there are slight variations in certain steps, we followed a similar methodology and used the same reagents described above in *Single-genome sequencing and cloning of gp160* *env* *genes for Part A*. To extract RNA, virions were concentrated from plasma or serum. In the RT step, the sequence of the gene-specific RT primer was CCCGCGTGGCCTCCTGAATTATNNNNNNNNGTCATTGGTCTTAAAGGTACCTG (CCCGCGTGGCCTCCTGAATTAT: reverse primer binding site for PCR amplification, NNNNNNNN: 8-base UMI, GTCATTGGTCTTAAAGGTACCTG: RT primer binding to RNA template). In the high-throughput single-genome sequencing (HT-SGS), we added cDNA purification step, in which the cDNA was purified with a 2.2:1 volumetric ratio of RNAClean XP solid phase reverse immobilization beads (A63987, Beckman Coulter). We followed the same previously described downstream process for HT-SGS (39). The purified cDNAs were amplified using the Advantage 2 PCR kit (639206, Takara Bio) with forward (GAGCAGAAGACAGTGGCAATGA) and reverse (CCCGCGTGGCCTCCTGAATTAT) primers (initial denaturation at 95°C for 1 minute, 31 cycles of denaturation at 95°C for 10 seconds, annealing at 64°C for 30 seconds, extension at 68°C for 3 minutes, and final extension at 68°C for 10 minutes). Libraries of amplified DNA products with a size of 3 kb (2.5 kb *env* gene) were constructed using the SMRTbell Express Template Prep Kit 2.0 (100-938-900, Pacific Biosciences), along with the Barcoded Overhang Adapter kit 8A and 8B (101-628-400 and 101-628-500, Pacific Biosciences) for multiplex sequencing. In the sequencing preparation step, the Sequel II Binding Kit 2.0 (101-842-900, Pacific Biosciences) was used for primer annealing, polymerase binding, and complex clean-up. Finally, the treated multiplexed samples were sequenced by a Sequel II system (Pacific Biosciences) with a 30-hour movie time under circular consensus sequencing (CCS) mode. Representative genes for functional studies were identified using Longitudinal Antigenic Sequences and Sites from Intra-Host Evolution (LASSIE) (40). LASSIE was run comparing pre- and posttreatment sequences to the most frequent sequence in the pretreatment sequences and selecting representative sequences with a variant count between 5 and 20. *Env* genes were synthesized by Twist Bioscience using a *Homo* *sapiens* codon table for codon optimization. They were cloned into the expression vector pTwist CMV BG WPRE Neo with the insertion points at Xhol/Xbal.

### Neutralization assays for sensitivity to VRC01LS and VRC07-523LS.

Neutralization of viruses by bNAbs was measured using single-round HIV-1 ENV-pseudovirus infection of TZM-bl target cells as previously described (41, 42). Pseudovirus stocks were generated by cotransfecting 293T cells (ATCC, CRL-3216) with the ENV plasmid and an ENV-deficient backbone (pSG3 ΔENV) at a 1:3 ratio by mass of DNA for Part A or at a 1:30 ratio for codon-optimized *env*s from Part B. At 72 hours after transfection, culture supernatants were filtered, harvested, and frozen at −80°C until further use. In the neutralization assay, 10 μL of 5-fold serially diluted bNAb (VRC01LS, VRC07-523LS, 3BNC117, and non-CD4bs 10E8) was incubated with 40 μL of pseudovirus in a 96-well plate at 37°C for 30 minutes before addition of TZM-bl cells (NIH AIDS Reagent Program, 8129). After 2 days of incubation, cells were lysed, and the viral infectivity was quantified by measuring luciferase activity with a SpectraMax Glo Steady-Luc Reporter Assay Kit (Molecular Devices). Each virus-antibody pairing was performed in duplicate wells. Neutralization curves were calculated by averaging duplicate wells and comparing luciferase units of wells containing antibody to virus-only controls after background subtraction. Curves were fit by nonlinear regression using the asymmetric 5-parameter logistic equation in Prism 9 for macOS (GraphPad Software, LLC). IC_50_ and IC_80_ are estimates of the antibody concentrations required to inhibit infection by 50% and 80%, respectively.

### Outcomes.

The primary objective of the study was to evaluate the safety and tolerability of VRC01LS or VRC07-523LS administered i.v. to PWH at a single dose of 40 mg/kg. The secondary objective was to assess the PK of VRC01LS and VRC07-523LS for 48 weeks following a single i.v. administration, the effect on VL and CD4^+^ T cell count, and determine whether ADA were present after administration.

### Statistics.

The determination of the sample size was based on the probability of observing an SAE, as well as the precision of SAE rate estimation represented by the 95% exact confidence intervals around the series of possible true SAE rates.

Maximum decline in log_10_ VL was calculated for each participant from the baseline visit to the time point corresponding to the minimum VL measured before ART initiation. The mean, median, and SD of maximum decline in log_10_ VL were computed for participants in Part A and Part B, respectively. Similarly, the mean, median, and SD of the maximum increase in CD4^+^ T cell counts were summarized among participants in Part A and Part B, respectively. To evaluate the relationship between the maximum log_10_ VL decline and baseline IC_80_, participants from Parts A and B were combined, and Spearman’s correlation was used. Furthermore, Wilcoxon’s rank-sum test was conducted to compare the distribution of maximum log_10_ VL decline between participants in Part A and those in Part B before the initiation of ART, and Wilcoxon’s signed-rank test was used to compare baseline CD4^+^ T cell counts to peak CD4^+^ T cell counts by day 14 prior to ART. PT_80_ values were calculated by dividing C_max_ by baseline PhenoSense IC_80_. Correlation between PT_80_ and maximum log_10_ VL was computed using Spearman’s correlation. PK parameters were compared using a 2-tailed *t* test. Statistical significance was assumed at a *P* value of less than 0.05. All analyses were performed using R version 4.1.3.

### Study approval.

The study protocol was reviewed and approved by NIAID, ACTG, and UPenn Institutional Review Boards (IRB). All study participants provided written informed consent prior to study enrollment.

### Data availability.

Results generated from this study are available as deidentified data on ClinicalTrials.gov (NCT02840474) and the raw data are in the Supporting Data Values file. The study protocol and informed consent form are also available on ClinicalTrials.gov. Additional data may be made available upon request to the corresponding author for investigators whose proposed use of the data has been approved by the NIH IRB.

## Author contributions

RML, CJF, SLH, SLK, RJL, RMP, JLSB, LAH, LN, MCC, EAB, EVC, EEC, RAK, JEL, JRM, GLC, and PT conceptualized the study and designed the methodology. CJF, SLH, SLK, RJL, RMP, JLSB, RLT, LAH, LN, JEL, GLC, and PT performed the clinical investigation. RML, LAH, LN, AMB, BD, SHK, FB, AMN, JPP, RC, MB, LS, SRN, BCL, ABM, and PT performed data collection. MH, RML, CJF, SLH, SLK, RJL, RMP, JLSB, RLT, LAH, LN, LS, JW, ZH, AMB, BD, SHK, FB, AMN, JPP, RC, MB, LS, SRN, BCL, ABM, EAB, EVC, EEC, GLC, and PT analyzed and interpreted the trial data. MH, RML, RLT, LS, BD, EEC, and PT visualized the trial data. RAK, JEL, JRM, GLC, and PT obtained funding for the study. MH, RML, CJF, SLH, SLK, RJL, RMP, JLSB, LAH, LN, JCR, RSR, ZH, MCC, LS, SRN, BCL, ABM, EAB, EEC, JEL, GLC, and PT performed project administration and supervisory roles. MH, RML, CJF, SLH, SLK, RJL, RMP, RLT, LS, JW, ZH, SHK, EVC, EEC, and PT wrote the original draft of the manuscript. MH, RML, CJF, SLH, SLK, RJL, RMP, JLSB, RLT, LAH, LN, JCR, RSR, LS, JW, ZH, MCC, AMB, BD, SHK, FB, AMN, JPP, RC, MB, LS, SRN, BCL, ABM, EAB, EVC, EEC, RAK, JEL, JRM, GLC, and PT reviewed and provided edits to the manuscript. MH and RML contributed equally to this work; the authorship order was determined alphabetically. All authors critically reviewed and approved the final submission.

## Supplementary Material

Supplemental data

ICMJE disclosure forms

Supporting data values

## Version 1 02/24/2025

Electronic publication
