# Supplementary material for: Virologic effects of broadly neutralizing antibodies VRC01LS and VRC07-523LS on chronic HIV-1 infection
Source: JCI Insight. 2025 Feb 24;10(4):e181496. doi: 10.1172/jci.insight.181496 (PMC11949028; doi:10.1172/jci.insight.181496)
Supplement: Supplemental data [file jciinsight-10-181496-s127.pdf]

Supplementary Materials for

Virologic effects of broadly neutralizing antibodies VRC01LS and VRC07-523LS on chronic HIV-1 infection

Myra Happe, Rebecca Lynch *et al.*

Corresponding author: Emily E. Coates, emily.coates@nih.gov

The PDF file includes:

Materials and Methods
Fig S1
Table S1 to S11

Supplementary Materials and Methods:

Participant IgG1 genetic marker allotyping

To assess allotype-specific effects on the PK parameters of VRC01LS and VRC07-523LS, study participants were evaluated for their GM 3/17 allotypes, specifically IgG1 GM 3/f and 17z (arginine to lysine). This analysis was performed as previously described (*7*).

Antidrug antibody assay (ADA)

A three-level tiered approach consisting of screening (tier 1), confirmation (tier 2), and functional characterization (tier 3) assays was utilized for ADA testing as previously described (*7, 8*). Briefly, tier 1 screening assays were performed using a Meso Scale Discovery (MSD) Electrochemiluminescence (ECLIA) homogeneous bridging assay (*7, 8*). Serially diluted serum samples were incubated with a reporter SULFO-TAG mAb and biotinylated capture mAb and then added to streptavidin-coated MSD plates. Any ADA present in the serum bound both the biotinylated and SULFO-TAG-labelled therapeutic mAbs forming a bridging complex attached to streptavidin-coated MSD plate. Luminescence intensity (ECL) was measured by MSD plate reader. Samples with ECL greater than the floating positivity cut-off point were considered tier 1-positive and used for tier 2 analysis. These samples were pre-incubated with or without the unlabeled VRC01LS or VRC07-523LS and were evaluated for the reduction in signal in the presence of unlabeled VRC01LS or VRC07-523LS (tier 2 assay). If samples displayed the % reduction of signal greater than positivity cut-off point, they were advanced to further testing. In this study, none of the collected samples yielded a positive tier 2 signal, therefore HIV

neutralization assay (tier 3) using pseudovirus with an antiretroviral (ARV) resistant backbone to functionally characterize the ADA was not performed.

Neutralization assay

The neutralization activity of serum samples collected from all study participants was assessed by utilizing an automated workstation consisting of Beckman Coulter Biomek FX liquid handling system equipped with 96 and 384 pipette tip heads, Thermo Cytomat Ambient Hotel, Thermo Cytomat 37°C incubator, and Molecular Devices Paradigm Multi-Mode detection platform with a luminescence cartridge. Briefly, serially diluted serum samples were incubated with an optimized concentration of a panel of HIV pseudoviruses representing diverse env sequences with known sensitivity to CD4 binding site antibody neutralization: CAP210.2.00.E8 (subtype C), MW965.26 (subtype C), PVO.04 (subtype B), Q23.env.17 (subtype A), THRO.18 (subtype B), and Simian Immunodeficiency virus, Macaque strain 251 (SIV_Mac251.30), which used as negative control. Pseudoviruses were incubated with serum for 45-60 minutes at 37°C prior to adding TZM-BL luciferase reporter cells (Bei Resources, cat. No ARP-8129) and incubated for an additional 48-54 hours. Following incubation, luciferase substrate was added, and the relative luminescence signal (RLU) was measured by Paradigm luminometer. The neutralization activity was quantified by computing the percentage of reduction in RLU of the samples against RLU range of assay control (Range = virus controls-cell controls). The neutralization percentages of each sample were plotted on a 5-parameter non-linear regression curve plot interpolated with corresponding ID_{50} and ID_{80} (50% and 80% neutralization titer).

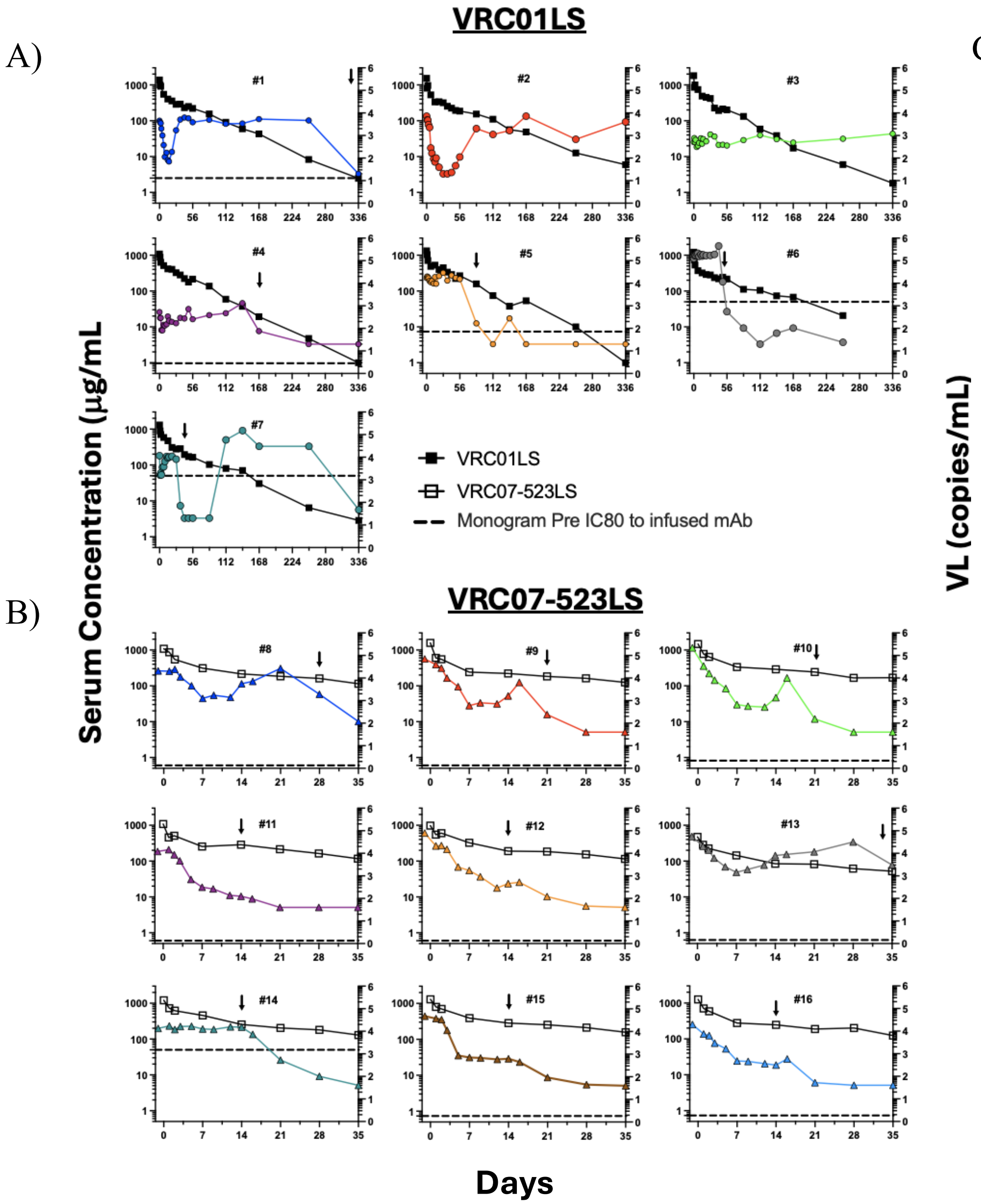

C)

Participant	PT80
1	528.1
2	.
3	754.6
4	1317.8
5	276.4
6	24.4
7	26.1
8	2631.02
9	7001.3
10	1797.9
11	2871.4
12	6533.5
13	757.1
14	27.4
15	1782.5
16	1932.3

Fig. S1. Viral load kinetics in relation to circulating antibody concentration and baseline virus sensitivity. Plasma viral load (right y-axis) and serum concentration (left y-axis) of VRC01LS (A) and VRC07-523LS (B) are displayed over time for each study participant. Arrows indicate study visit at which participants self-reported antiretroviral therapy (ART) usage. Arrows do not necessarily represent date of ART initiation. Horizontal dotted line indicates the pre-infusion IC_{80} to infused mAb estimated by Monogram PhenoSense mAb assay for each participant. C) PT_{80} was calculated based on maximum serum concentration (C_{max}) values and baseline IC_{80} to infused mAb estimated by Monogram PhenoSense mAb assay (C_{max}/IC_{80}). The baseline IC_{80} for participant 2 was unable to be measured.

Table S1. Demographic characteristics of study participants.

Category	Characteristic	VRC01LS 40 mg/kg IV (n=7)	VRC07-523LS 40 mg/kg IV (n=9)	Overall (n=16)
			n (%)	
Sex	Male	5 (71.4)	9 (100.0)	14 (87.5)
	Female	2 (28.6)	0 (0)	2 (12.5)
Age (Years)	18-20	0 (0)	2 (22.2)	2 (12.4)
	21-30	3 (42.9)	4 (44.4)	7 (43.8)
	31-40	1 (14.3)	2 (22.2)	3 (18.8)
	41-50	1 (14.3)	0 (0)	1 (6.2)
	51-60	2 (28.6)	1 (11.1)	3 (18.8)
	Mean (SD)	39.9 (13.9)	28.8 (11.0)	33.6 (13.2)
	Median [Range]	39 [25, 57]	26 [20, 56]	27.5 [20, 57]
Race	White	2 (28.6)	7 (77.8)	9 (56.3)
	Black or African American	5 (71.4)	1 (11.1)	6 (37.5)
	Unknown/Not Reported	0 (0)	1 (11.1)	1 (6.2)
Ethnicity	Non-Hispanic/Latino	7 (100.0)	7 (77.8)	14 (87.5)
	Hispanic/Latino	0 (0)	2 (22.2)	2 (12.5)
Weight (kilograms)	Mean (SD)	91.2 (22.3)	81.4 (19.4)	85.7 (20.6)
	Median [Range]	77.4 [69.1, 124.6]	85.2 [51.7, 110.5]	84.6 [51.7, 124.6]
HIV Treatment	Not on ART Treatment	7 (100.0)	9 (100.0)	16 (100.0)

Table S2. Clinical characteristics of study participants.

Participant	Years since HIV diagnosis	ART status at baseline	Baseline HIV VL (copies/mL)	Baseline CD4 (cells/mcL)	ART status change[$] (days post infusion) /Regimen	IgG1 GM Allotype	Product (40 mg/kg IV)
1	9	Naïve	3177	543	336/ FTC, TDF, EVG	3/17	VRC01LS
2	1	Naïve	6394	576	No ART Therapy	17/17	VRC01LS
3	13	Naïve	499	509	No ART Therapy	3/17	VRC01LS
4	8	Naïve	258	817	No ART Therapy*	17/17	VRC01LS
5	0.65	Naïve	12508	395	84/ FTC, TDF, EVG	17/17	VRC01LS
6	0.7	Naïve	174134	418	49/ FTC, TDF, EVG	17/17	VRC01LS
7	2	Off	874	523	35/ ABC, 3TC, DTG	17/17	VRC01LS
8	0.8	Naïve	20738	525	28/ TAF, FTC, BIC	3/17	VRC07-523LS
9	0.8	Naïve	70435	382	21/ TAF, FTC, EVG	3/17	VRC07-523LS
10	0.8	Naïve	65708	827	21/ TAF, FTC, BIC	17/17	VRC07-523LS
11	1	Naïve	12149	733	14/ TAF, FTC, BIC	3/17	VRC07-523LS
12	1	Naïve	41906	533	14/ TAF, FTC, BIC	17/17	VRC07-523LS
13	1.1	Naïve	19828	754	35/ TAF, FTC, BIC	3/17	VRC07-523LS
14	1.1	Naïve	14402	448	14/ TAF, FTC, BIC	3/17	VRC07-523LS
15	1.1	Naïve	41622	542	14/ TAF, FTC, BIC	3/17	VRC07-523LS
16	1	Naïve	15949	430	14/ TAF, FTC, BIC	3/17	VRC07-523LS

ART= Antiretroviral therapy: Abacavir (ABC), Bictegravir (BIC), Dolutegravir (DTG), Elvitegravir (EVG), Emtricitabine (FTC), Lamivudine (3TC), Tenofovir alafenamide (TAF), Tenofovir (TDF).
VL= Viral load. IV= Intravenous.
*Participant tested positive for ART (EVG, FTC, and TFV) on day 168 but did not report use of ART in the study.
[$]Study day reflects only the date the self-reported ART data were collected and does not necessarily represent an ART start date.

Table S3. Maximum local and systemic solicited reactogenicity.

	VRC01LS 40 mg/kg IV (n=7)	VRC07-523LS 40 mg/kg IV (n=9)	Overall (n=16)
**Local Symptom**		n (%)	
Bruising			
None	7 (100.0)	7 (77.8)	14 (87.5)
Mild	0 (0)	2 (22.2)	2 (12.5)
Pain/Tenderness			
None	7 (100.0)	6 (66.7)	13 (81.3)
Mild	0 (0)	3 (33.3)	3 (18.7)
Any Local Symptom			
None	7 (100.0)	6 (66.7)	13 (81.3)
Mild	0 (0)	3 (33.3)	3 (18.7)
**Systemic Symptom**		n (%)	
Chills			
None	6 (85.7)	9 (100.0)	16 (100.0)
Mild	1 (14.3)	0 (0)	0 (0)
Headache			
None	6 (85.7)	8 (88.9)	14 (87.5)
Mild	1 (14.3)	1 (11.1)	2 (12.5)
Malaise			
None	7 (100.0)	7 (77.8)	14 (87.5)
Mild	0 (0)	2 (22.2)	2 (12.5)
Myalgia			
None	7 (100.0)	8 (88.9)	15 (93.8)
Mild	0 (0)	1 (11.1)	1 (6.2)
Nausea			
None	6 (85.7)	9 (100.0)	15 (93.8)
Mild	1 (14.3)	0 (0)	1 (6.2)
Any Systemic Symptom			
None	6 (85.7)	7 (77.8)	13 (81.3)
Mild	1 (14.3)	2 (22.2)	3 (18.7)

Table S4. Antidrug antibody analysis.

Study group	Participant	Time Point	Tier 1	Tier 2	Tier 1	Tier 2
**VRC01LS 40 mg/kg IV**	1	Pre-treatment	Negative	N/A		
		4 weeks post	Negative	N/A		
		8 weeks post	Negative	N/A		
	2	Pre-treatment	Negative	N/A		
		4 weeks post	Negative	N/A		
		8 weeks post	Negative	N/A		
	3	Pre-treatment	Negative	N/A		
		4 weeks post	Negative	N/A		
		8 weeks post	Negative	N/A		
	4	Pre-treatment	Negative	N/A		
		4 weeks post	Negative	N/A		
		8 weeks post	Negative	N/A		
	5	Pre-treatment	Positive	Negative		
		4 weeks post	Negative	N/A		
		8 weeks post	Negative	N/A		
	6	Pre-treatment	Negative	N/A		
		4 weeks post	Negative	N/A		
		8 weeks post	Negative	N/A		
	7	Pre-treatment	Negative	N/A		
		4 weeks post	Negative	N/A		
		8 weeks post	Negative	N/A		
**VRC07-523LS 40 mg/kg IV**	8	Pre-treatment			Negative	N/A
		4 weeks post			Negative	N/A
		8 weeks post			Negative	N/A
	9	Pre-treatment			Negative	N/A
		4 weeks post			Negative	N/A
		8 weeks post			Negative	N/A
	10	Pre-treatment			Positive	Negative
		4 weeks post			Negative	N/A
		8 weeks post			Negative	N/A
	11	Pre-treatment			Negative	N/A
		4 weeks post			Negative	N/A
		8 weeks post			Negative	N/A
	12	Pre-treatment			Negative	N/A
		4 weeks post			Negative	N/A
		8 weeks post			Negative	N/A
	13	Pre-treatment			Negative	N/A
		4 weeks post			Positive	Negative
		8 weeks post			Positive	Negative
	14	Pre-treatment			Negative	N/A
		4 weeks post			Negative	N/A
		8 weeks post			Negative	N/A
	15	Pre-treatment			Negative	N/A
		4 weeks post			Negative	N/A
		8 weeks post			Negative	N/A
	16	Pre-treatment			Negative	N/A
		4 weeks post			Negative	N/A
		8 weeks post			Negative	N/A

Tier 3 ADA testing was not performed because all tier 2 results were negative.

Table S5. VRC01LS and VRC07-523LS mean PK parameter values.

Study group	C_{max} mg/mL	T_{max} (hours)	CL (mL/d)	V_d (L)	$t_{1/2}$ (days)	AUC_{last} (mg*d/mL)	C_{14D} (mg/mL)	C_{84D} (mg/mL)
				Mean (SD)				
**VRC01LS** 40 mg/kg IV (n=7)	1566 (316)	1.7 (1.4)	104.3 (21.4)	7.07 (1.7)	47.3 (8.4)	34280 (2796)	423 (76)	136.2 (21.9)
**VRC07-523LS** 40 mg/kg IV (n=9)	1295 (376)	1.4 (1.2)	188.7 (55.8)	15.6 (6.5)	56.5 (13.2)	17967 (4563)	231 (65)	60.7 (20.8)

C_{max}= maximum concentration, T_{max}= time to maximum concentration, CL=clearance, V_d= volume of distribution. $t_{1/2}$ =half-life, AUC= area under the curve, C_{14D}, C_{84D}= concentration on day 14 and 84, respectively. SD stands for standard deviation.

Table S6. Post-infusion functionality of administered VRC01LS and VRC07-523LS.

Study group	Participant	Days post infusion	CAP210.E8	MW965.26	PVO.04	Q23.17	THRO.18
			ID_{80}	ID_{80}	ID_{80}	ID_{80}	ID_{80}
**VRC01LS 40 mg/kg IV**	**1**	0	<10.0	2117.98	<10.0	<10.0	<10.0
		2	<10.0	10681.92	646.39	4021.31	45.56
	**2**	0	<10.0	1670.92	<10.0	<10.0	<10.0
		2	<10.0	7562.03	481.25	2867.4	51.76
	**3**	0	<10.0	372.74	66.53	<10.0	17.79
		2	<10.0	3156	409.67	1649.55	56.32
	**4**	0	<10.0	22.42	<10.0	<10.0	<10.0
		2	<10.0	3342.58	336.24	1269.09	29.95
	**5**	0	<10.0	53.26	<10.0	<10.0	<10.0
		2	<10.0	5637.43	509.03	1946.29	26.55
	**6**	0	<10.0	22.63	<10.0	<10.0	<10.0
		2	<10.0	2477.8	173	1247.38	30.81
	**7**	0	17.68	631.7	<10.0	29.2	<10.0
		2	12.58	4648.04	339.78	1369.15	53.86
**VRC07-523LS 40 mg/kg IV**	**8**	0	<20.0	<20.0	<20.0	<20.0	<20.0
		14	201.31	12354.31	1518.57	4183.38	172.65
	**9**	0	<20.0	<20.0	<20.0	<20.0	<20.0
		14	180.02	10999.14	1731.35	3965.97	152.15
	**10**	14	198.53	13111.86	1246.12	4185.68	156.14
	**11**	0	<20.0	67.42	<20.0	<20.0	<20.0
		14	111.82	12657.43	1698.98	4633.83	167.93
	**12**	0	<20.0	<20.0	<20.0	<20.0	<20.0
		14	80.74	6337.79	904.15	2359.03	77.97
	**13**	0	<20.0	<20.0	<20.0	<20.0	<20.0
		14	172.62	15148.03	2848.64	6387.66	191.32
	**14**	0	<20.0	547.32	<20.0	<20.0	<20.0
		14	155.66	19050.53	1459.71	5055.92	261.27
	**15**	0	<20.0	506.24	<20.0	<20.0	<20.0
		14	216.39	12727.80	1911.93	5139.92	261.19
	**16**	0	<20.0	<20.0	<20.0	<20.0	<20.0
		14	117.58	17556.31	2274.16	7599.95	207.09

Table S7. Characteristics of virus load kinetics.

Study group	Participant	Baseline VL (log_{10})	Minimum VL prior to ART (log_{10})	Day of minimum log_{10} VL	Maximum decline in VL (baseline to day 14, log_{10})
VRC01LS 40 mg/kg IV	1	3.6	1.9	14	-1.7
	2	3.9	1.0	14	-2.0
	3	2.7	2.5	5	-0.2
	4	2.5	1.9	5	-0.6
	5	4.2	4.0	12	-0.2
	6	5.1	5.2	9	+0.1
	7	3.8	3.2	1	-0.6
		Mean (SD); Median			
		3.7 (0.9); 3.8	2.9 (1.3); 2.5	8.6 (5.1); 9	-0.8 (0.8); -0.6
VRC07-523LS 40 mg/kg IV	8	4.3	3.1	7	-1.2
	9	4.9	2.8	7	-2.1
	10	5.3	2.7	12	-2.6
	11	4.1	2.1	14	-2.0
	12	4.9	2.5	12	-2.4
	13	4.8	3.2	7	-1.6
	14	4.1	4.1	2	-0.1
	15	4.7	2.8	12	-1.9
	16	4.3	2.5	14	-1.8
		Mean (SD); Median			
		4.6 (0.4); 4.7	2.8 (0.6); 2.8	9.7 (4.1); 12	-1.7 (0.8); -1.9

Changes in VL were calculated pre-ART from baseline to Day 14 post VRC01LS or VRC07-523LS administration. SD stands for standard deviation.

Table S8. Changes in CD4 counts.

Study group	Baseline CD4, cells/mcL	Peak CD4 post baseline to day 14 (prior to ART), cells/mcL	Day of peak	Increase in CD4, baseline to peak
		Mean (SD); Median		
**VRC01LS** 40mg/kg IV (n=7)	540 (138.6); 523	603 (236.4); 539	12 (3.4); 14	63 (139.3); 59
**VRC07-523LS** 40mg/kg IV (n=9)	567 (165.1); 525	694 (233.9); 622	10 (3.7); 7	128 (152.5); 82

Peak CD4 counts were calculated pre-ART from baseline to Day 14 post VRC01LS or VRC07-523LS administration. Fold change was the increase of CD4 count divided by the baseline CD4 count. .SD stands for standard deviation.

Table S9. PhenoSense population viral sensitivity pre- and post-infusion.

Study group	Participant	Days post infusion	IC_{50} VRC01LS	IC_{50} VRC07-523LS	IC_{50} 10E8V4	IC_{80} VRC01LS	IC_{80} VRC07-523LS	IC_{80} 10E8V4
VRC01LS 40 mg/kg IV	1	0	0.693	0.308	1.91	2.95	0.943	11.8
		28	>50	0.409	1.65	>50	1.91	12.7
		56	>50	0.407	1.19	>50	3.04	11.3
	2	0	~	~	~	~	~	~
		35	~	~	~	~	~	~
	3	0	~	~	~	~	~	~
		28	~	~	~	~	~	~
		56	0.360	0.209	0.892	2.41	0.517	3.50
	4	0	0.252	0.036	0.267	0.962	0.136	1.50
		28	0.861	0.574	0.432	21.4	7.47	2.44
		56	4.48	0.661	0.499	>50	14.6	2.04
	5	0	1.34	0.194	0.841	7.36	0.623	3.76
		28	2.96	0.183	0.774	12.0	0.752	4.72
	6	0	>50	0.205	0.382	>50	0.865	1.63
		28	>50	0.236	0.352	>50	1.85	2.28
	7	0	6.51	0.222	0.745	>50	1.26	3.57
		28	1.01	0.085	0.842	>50	0.325	3.68
VRC07-523LS 40 mg/kg IV	8	0	0.592	0.145	0.534	2.35	0.519	1.97
		7	0.659	0.120	0.545	2.76	0.444	2.40
		28	>50	4.01	0.654	>50	>50	3.00
	9	0	0.242	0.092	0.124	0.840	0.234	0.691
		7	.	.	.	.	.	.
	10	0	2.47	0.248	0.637	11.3	0.815	2.43
		7	3.37	0.215	0.981	10.8	0.995	3.81
	11	0	0.554	0.091	1.08	2.27	0.378	5.88
		7	0.855	0.145	1.50	3.06	0.252	7.06
	12	0	0.268	0.042	0.446	1.12	0.182	2.75
		7	0.266	0.058	0.385	1.05	0.187	1.82
	13	0	0.479	0.169	>50	2.32	0.627	>50
		7	0.740	0.334	>50	2.49	0.977	>50
		28	0.615	0.249	>50	2.12	1.09	>50
	14	0	>50	>50	0.655	>50	>50	3.22
		7	>50	>50	0.572	>50	>50	2.58
	15	0	13.8	0.23	0.614	>50	0.733	3.35
		7	.	.	.	.	.	.
		28	.	.	.	.	.	.
	16	0	0.696	0.234	0.762	2.59	0.820	5.03
		7	0.603	0.161	0.606	2.87	0.533	3.93

mAb concentrations µg/mL

<0.1	0.1-1.0	1.0-10.0	10.0-50.0

Table S10. Single clone virus sensitivity pre- and post-VRC01LS infusion to four HIV-1 monoclonal antibodies.

			IC_{50}				IC_{80}			
Participant	Days post infusion	Virus	VRC01LS	VRC07-523LS	3BNC117	10E8	VRC01LS	VRC07-523LS	3BNC117	10E8
1	0	1.00.B3	0.907	0.398	0.221	0.675	3.08	1.05	0.70	4.14
		1.00.A4	1.21	0.337	0.332	11.2	3.80	0.85	0.82	>50
		1.00.B7	2.37	0.633	0.564	0.420	7.40	1.55	1.75	2.47
		1.00.A8	4.02	1.11	0.931	0.266	15.4	3.57	2.78	2.27
		1.00.D4	1.48	0.351	0.351	0.375	4.86	0.94	1.19	2.42
	35	1.35.C5	>50	3.10	46.9	0.349	>50	20.5	>50	1.67
		1.35.A3	>50	1.42	40.0	0.234	>50	6.57	>50	1.93
		1.35.D11	9.31	1.96	1.91	0.405	22.4	4.25	5.15	2.64
		1.35.B1	>50	0.254	2.79	0.002	>50	1.66	25.0	0.76
		1.35.A9	>50	0.613	36.8	0.735	>50	2.54	>50	4.14
2	0	2.00.D6	0.371	0.207	0.195	4.77	0.75	0.41	0.42	13.3
		2.00.A10.3	0.554	0.274	0.145	1.00	1.50	0.81	0.41	5.11
		2.00.D10	0.674	0.450	0.332	1.28	1.99	1.63	1.41	5.66
		2.00.B12	0.839	0.581	0.372	2.17	2.48	1.90	0.96	2.17
		2.00.A8	0.558	0.267	0.254	1.11	1.60	0.80	0.44	14.6
		2.00.A11.2	1.54	0.378	0.687	0.176	4.09	1.96	2.32	1.06
	35	2.35.A8	6.11	>50	2.87	0.546	>50	>50	>50	2.57
		2.35.D11	9.85	7.36	>50	>50	>50	>50	>50	>50
		2.35.B12	11.2	6.11	>50	1.94	>50	>50	>50	8.59
		2.35.A7.2	5.62	2.43	>50	0.946	>50	23.8	>50	5.56
3	0	3.00.A122	>50	0.090	>50	0.110	>50	0.320	>50	0.380
		3.00.A62	3.90	0.920	>50	0.150	30.6	3.20	>50	0.680
		3.00.B12	4.14	1.82	5.12	0.170	9.89	4.19	9.51	0.890
		3.00.C1	1.03	0.170	>50	0.263	3.05	0.400	>50	0.841
4	0	4.00.G9	>50	>50	>50	0.565	>50	>50	>50	1.62
		4.00.B11	0.609	0.105	>50	0.548	2.58	0.448	>50	2.08
		4.00.B9	0.280	0.061	>50	0.444	0.699	0.156	>50	1.25
		4.00.B12	1.57	0.282	>50	0.509	6.43	2.23	>50	1.72
	28	4.28.B6	>50	42.0	>50	0.225	>50	>50	>50	0.852
		4.28.E12	0.897	0.157	>50	0.262	4.19	0.992	>50	1.30
		4.28.E2.2	1.30	0.202	>50	0.141	17.0	7.94	>50	0.815
		4.28.F2	>50	28.1	>50	0.205	>50	>50	>50	1.05
		4.28.A11	>50	>50	>50	0.149	>50	>50	>50	1.06
5	0	5.00.C1	11.7	1.38	1.18	0.965	30.6	2.50	2.78	2.97
		5.00.E2	6.40	1.31	0.764	0.475	15.9	1.31	1.84	1.62
		5.00.G2	13.3	1.52	1.59	3.45	29.5	3.09	3.29	8.85
		5.00.G3	6.00	0.524	0.723	0.505	15.6	1.47	2.22	1.83
	28	5.28.A3	7.63	0.627	0.411	0.212	>50	2.03	2.60	0.817
		5.28.C7	19.0	1.30	1.65	1.59	48.2	3.16	4.60	4.73
		5.28.G4	12.3	1.04	2.25	0.695	32.0	2.51	5.50	3.14
		5.28.G12	19.4	1.28	2.77	1.11	>50	3.45	7.13	2.98
		5.28.B10	27.9	2.38	1.17	0.253	>50	3.31	7.79	1.31
6	0	6.00.A11	>50	0.695	>50	0.124	>50	4.87	>50	1.31
		6.00.C10	>50	0.641	>50	0.204	>50	3.50	>50	1.01
		6.00.D12	>50	0.220	>50	0.554	>50	0.682	>50	2.49
		6.00.F12	>50	0.703	>50	0.016	>50	3.56	>50	0.597
		6.00.G5	>50	0.261	>50	0.044	>50	4.16	>50	0.443
	28	6.28.C10	>50	>50	>50	>50	>50	0.368	>50	0.058
		6.28.G4	>50	1.06	>50	0.126	>50	7.60	>50	0.869
		6.28.H7	>50	0.883	>50	0.241	>50	5.12	>50	1.29
		6.28.H10	>50	1.08	>50	0.083	>50	11.9	>50	0.875

mAb concentrations μg/mL

<0.1	0.1-1.0	1.0-10.0	10.0-50.0

Table S11. Single clone virus sensitivity pre- and post-VRC07-523LS infusion to four HIV-1 monoclonal antibodies.

			IC_{50}				IC_{80}			
Participant	Days post infusion	Virus	VRC01LS	VRC07-523LS	3BNC117	10E8	VRC01LS	VRC07-523LS	3BNC117	10E8
8	0	8.00.B1	3.09	0.659	4.34	0.288	9.38	2.21	9.99	1.12
		8.00.B2	1.99	0.465	21.1	0.474	7.31	1.40	>50	1.69
		8.00.B3	2.53	0.679	2.83	0.472	10.5	2.23	12.1	1.57
		8.00.B4	2.55	0.269	3.10	0.367	7.51	0.862	9.32	1.55
		8.00.B5	8.04	1.81	7.88	2.48	17.7	2.95	16.7	6.98
		8.00.B6	4.31	0.781	6.50	1.08	13.5	2.10	15.2	3.93
	14	8.14.B7	>50	9.75	>50	1.11	>50	>50	>50	2.92
		8.14.B8	>50	>50	>50	1.59	>50	>50	>50	3.95
		8.14.B9	>50	>50	>50	0.514	>50	>50	>50	1.98
		8.14.B10	>50	>50	>50	1.01	>50	>50	>50	3.16
		8.14.B11	>50	>50	>50	2.44	>50	>50	>50	5.28
9	0	9.00.C2	0.113	0.144	0.867	0.019	0.403	0.641	2.12	0.162
		9.00.C3	0.410	0.099	0.132	0.077	1.20	0.420	0.368	0.724
		9.00.C4	1.28	0.588	0.362	0.177	2.76	1.27	0.666	0.688
		9.00.C5	1.80	0.627	0.415	0.325	3.75	1.24	0.874	1.01
		9.00.C6	1.25	0.088	0.277	0.592	2.39	0.383	0.442	3.77
		9.00.C7	1.56	0.443	0.274	0.133	3.43	0.945	0.656	0.479
		9.00.C8	0.527	0.275	0.230	0.108	1.17	0.442	0.454	0.342
		9.00.C9	1.28	0.319	0.177	0.054	2.25	0.631	0.415	0.396
		9.00.C10	0.782	0.261	0.117	0.027	1.72	0.719	0.311	0.132
	14	9.14.A9	0.571	0.202	0.165	0.018	1.72	0.421	0.418	0.200
		9.14.A10	>50	>50	>50	0.066	>50	>50	>50	0.327
		9.14.A11	>50	3.01	0.366	0.077	>50	13.6	0.868	0.457
		9.14.A12	0.469	0.140	0.125	0.037	1.46	0.332	0.396	0.174
		9.14.A13	31.2	15.7	0.196	0.018	>50	>50	0.787	0.103
10	0	10.00.D1	17.1	0.576	>50	1.47	47.3	1.57	>50	4.52
		10.00.D2	16.6	1.24	>50	2.07	41.4	3.27	>50	5.76
		10.00.D3	9.81	1.06	>50	1.09	37.7	2.78	>50	3.51
		10.00.D4	9.92	0.930	>50	1.02	27.1	2.18	>50	2.84
		10.00.D5	13.2	1.52	>50	1.96	43.5	3.31	>50	5.76
	14	10.14.D8	10.9	1.36	>50	2.01	34.7	3.14	>50	5.02
		10.14.D9	>50	>50	>50	0.632	>50	>50	>50	4.34
11	0	11.00.E1	2.70	0.587	0.692	2.84	10.8	1.66	2.25	9.61
		11.00.E2	6.00	0.741	0.782	2.07	14.4	1.82	3.34	6.35
		11.00.E3	3.31	0.557	0.400	1.39	8.91	1.43	3.85	3.29
		11.00.E4	3.65	0.454	0.726	1.99	9.23	1.46	1.85	6.94
		11.00.E5	2.47	0.488	0.424	1.97	8.12	1.37	1.59	7.62
		11.00.E6	2.94	0.414	0.478	4.22	7.77	0.990	1.34	10.9
		11.00.E7	5.01	0.512	0.818	5.49	13.2	1.27	2.07	20.8
		11.00.E8	3.95	0.287	6.37	1.40	11.2	0.723	34.0	3.37
	14	11.14.A1	8.30	0.707	26.2	4.24	17.6	1.87	>50	9.22
		11.14.A2	3.16	0.353	0.647	2.70	7.80	1.33	1.72	7.88
		11.14.A3	2.16	0.214	2.87	1.11	5.03	0.578	12.7	2.69
		11.14.A4	4.24	0.345	1.64	0.259	16.7	1.45	4.34	1.77
12	0	12.00.F1	1.18	0.121	0.304	0.814	2.60	0.336	0.713	3.20
		12.00.F2	1.64	0.176	0.620	1.52	3.12	0.367	1.27	3.30
		12.00.F3	1.26	0.155	0.373	0.907	3.74	0.401	0.908	4.05
		12.00.F4	1.33	0.130	0.491	1.48	2.25	0.431	1.31	3.83

		12.00.F5	0.900	0.125	0.282	0.659	2.21	0.330	0.715	2.12
		12.00.F6	1.35	0.153	0.485	1.20	2.45	0.342	1.38	2.76
		12.00.F7	0.704	0.093	0.173	0.876	1.95	0.258	0.569	3.27
		12.00.F11	1.21	0.084	0.198	1.60	1.98	0.196	0.659	4.79
	14	12.14.F20*	1.33	0.130	0.491	1.48	2.25	0.431	1.31	3.83
		12.14.F21	1.26	0.155	0.373	0.907	3.740	0.401	0.908	4.05
		12.14.F22	0.704	0.093	0.173	0.876	1.95	0.258	0.569	3.27
		12.14.F13	1.48	0.077	0.205	2.29	5.84	0.224	0.916	8.07
13	0	13.00.G3	1.45	0.231	0.689	>50	2.77	0.809	1.98	>50
		13.00.G4	0.583	0.299	0.347	33.3	1.84	0.487	0.976	>50
		13.00.G5	0.664	0.425	0.724	>50	3.49	1.49	1.87	>50
		13.00.G6	0.531	0.240	0.314	38.3	1.53	0.474	0.914	>50
		13.00.G7	1.15	0.302	0.947	>50	2.05	0.941	1.88	>50
		13.00.G8	0.521	0.176	0.372	13.6	2.44	0.461	1.83	>50
		13.00.G9	0.721	0.147	0.366	>50	1.69	0.478	1.36	>50
	14	13.14.G10	1.27	0.340	1.12	>50	2.28	1.25	1.90	>50
		13.14.G11	1.23	0.601	0.751	2.64	3.02	1.57	1.93	2.88
		13.14.G12	1.16	0.465	0.679	39.3	2.13	1.22	1.26	>50
		13.14.G20	0.664	0.425	0.724	>50	3.49	1.49	1.87	>50
		13.14.G21	1.15	0.302	0.947	>50	2.05	0.941	1.88	>50
		13.14.G13	0.880	0.371	0.952	>50	2.41	0.820	1.99	>50
		13.14.G14	0.347	0.279	0.386	9.20	1.51	0.930	1.41	>50
14	0	14.00.H1	>50	>50	>50	0.055	>50	>50	>50	1.02
		14.00.H2	>50	>50	>50	0.420	>50	>50	>50	2.24
		14.00.H3	>50	>50	>50	0.177	>50	>50	>50	1.56
		14.00.H4	>50	>50	>50	0.546	>50	>50	>50	1.77
		14.00.H5	>50	>50	>50	2.19	>50	>50	>50	4.88
	14	14.14.H20	>50	>50	>50	0.420	>50	>50	>50	2.24
		14.14.H21	>50	>50	>50	0.177	>50	>50	>50	1.56
		14.14.H22	>50	>50	>50	0.546	>50	>50	>50	1.77
		14.14.H6	>50	>50	>50	1.19	>50	>50	>50	3.26
		14.14.H7	>50	>50	>50	0.636	>50	>50	>50	2.27
15	0	15.00.J1	33.9	0.615	>50	0.489	>50	1.52	>50	2.02
		15.00.J2	23.4	0.720	>50	0.538	>50	1.75	>50	1.75
		15.00.J5	15.9	0.576	>50	0.866	37.4	1.35	>50	3.09
		15.00.J6	6.42	0.357	1.97	0.393	28.8	1.28	5.20	1.64
		15.00.J7	0.971	0.214	0.678	0.529	5.27	0.527	2.23	1.97
		15.00.J10	>50	0.681	>50	0.233	>50	1.87	>50	1.47
		15.00.J12	>50	0.448	>50	0.208	>50	1.69	>50	1.89
		15.00.J13	19.5	0.585	>50	0.667	>50	1.32	>50	2.58
		15.00.J14	6.87	0.323	>50	0.224	29.8	0.926	>50	1.64
		15.00.J15	20.8	0.354	>50	0.417	>50	1.39	>50	2.03
		15.00.J16	45.4	0.374	>50	0.381	>50	1.04	>50	1.52
	14	15.14.A5	0.915	0.184	0.690	0.394	2.05	0.556	1.85	1.66
		15.14.A6	2.58	0.187	1.04	0.206	10.0	0.550	1.98	1.19
		15.14.A8	>50	0.234	>50	0.448	>50	2.35	>50	3.11

mAb concentrations μg/mL

<0.1	0.1-1.0	1.0-10.0	10.0-50.0

*Clones named in twenties represent sequences identical to those sampled pre-infusion

The VRC 607/ACTG A5378 Study Team: Study team members not listed in the author line include: Vaccine Research Center, NIAID, NIH: Maria Claudia Burgos Florez, Nina Berkowitz, Olga Vasilenko, Olga Trofymenko, Iris Pittman, Eugenia Burch, Lam Le, Somia Hickman, Katherine Houser, Cynthia Hendel, Sarah Plummer, Ingelise Gordon, Jamie Saunders, William Whalen, Pamela Costner, Kathy Zephir, Floreliz Mendoza, John H. Dohnal, Galina Yamshchikov, Christian Buettner, Mark O' Callahan, Carolyn Laurencot, KC Cheng, Judy Stein, Robert Bailer, Christopher Moore, John Rathmann, Clare Whittaker, Jagada Thillainathan, Britta Flach, and Alison Taylor; Division of Infectious Diseases, Washington University in St. Louis: Teresa Spitz, Michael Royal, Michael Klebert, and Lisa Kessels; Division of Infectious Diseases, The Ohio State University: Kathy Watson and Heather Harber; Department of Medicine, University of Pennsylvania: Eileen Donaghy, Rosemarie Kappes, and Deborah Kim; School of Medicine, University of Puerto Rico: Ileana Boneta Dueño and Sigrid Perez; Division of Infectious Diseases, University of Cincinnati: Linda Hinds and Marlena Petrie; Division of Infectious Diseases, David Geffen School of Medicine, University of California: Nicholas Pierce, Aleen Khodabakhshian, Ste'von Afemata, and Mary Catherine (Catie) Cambou.
